# Polycomb protein binding and looping in the ON transcriptional state

**DOI:** 10.1126/sciadv.adn1837

**Published:** 2024-04-24

**Authors:** J. Lesley Brown, Liangliang Zhang, Pedro P. Rocha, Judith A. Kassis, Ming-an Sun

**Affiliations:** ^1^ Eunice Kennedy Shriver National Institute of Child Health and Human Development, National Institutes of Health, Bethesda, MD 20892, USA.; ^2^ Institute of Comparative Medicine, College of Veterinary Medicine, Yangzhou University, Yangzhou, Jiangsu, China.; ^3^ National Cancer Institute, National Institutes of Health, Bethesda, MD 20892, USA.; ^4^ Jiangsu Co-innovation Center for Prevention and Control of Important Animal Infectious Diseases and Zoonosis, Joint International Research Laboratory of Agriculture and Agri-Product Safety of Ministry of Education of China, Yangzhou University, Yangzhou, Jiangsu, China.

## Abstract

Polycomb group (PcG) proteins mediate epigenetic silencing of important developmental genes by modifying histones and compacting chromatin through two major protein complexes, PRC1 and PRC2. These complexes are recruited to DNA by CpG islands (CGIs) in mammals and Polycomb response elements (PREs) in *Drosophila*. When PcG target genes are turned OFF, PcG proteins bind to PREs or CGIs, and PREs serve as anchors that loop together and stabilize gene silencing. Here, we address which PcG proteins bind to PREs and whether PREs mediate looping when their targets are in the ON transcriptional state. While the binding of most PcG proteins decreases at PREs in the ON state, one PRC1 component, Ph, remains bound. Further, PREs can loop to each other and with presumptive enhancers in the ON state and, like CGIs, may act as tethering elements between promoters and enhancers. Overall, our data suggest that PREs are important looping elements for developmental loci in both the ON and OFF states.

## Teaser

Polycomb response elements in the ON state can remain looped to each other and may also loop with putative active enhancers.

## INTRODUCTION

How organisms regulate gene expression as they develop from an egg to a complex organism is a fundamental question in biology. Early studies in the model organism *Drosophila* yielded a wealth of information about developmental genes and how they are regulated including the discovery of the Polycomb group (PcG) genes (*1*, *2*). The PcG genes encode a group of highly conserved proteins required for maintenance of the silenced state of important developmental genes (*3*–*5*). There are two main Polycomb-repressive complexes (PRCs) in both *Drosophila* and mammals, named PRC1 and PRC2, with both canonical and variant forms (*6*, *7*). PRC2 contains the histone methyltransferase E(z) in *Drosophila* (or EZH1/2 in mammals) that trimethylates histone H3 lysine 27 (H3K27me3), which covers repressed canonical PcG target genes. PRC1 can ubiquitinate H2AK118 in *Drosophila* (or H2AK119 in mammals) and can also compact chromatin. In addition to PRC1 and PRC2, *Drosophila* has two additional well-characterized PcG complexes: Pho-repressive complex (PhoRC) which is important for recruitment of PcG complexes to DNA and Polycomb-repressive deubiquitinase which deubiquitinates H2AK118ub (*8*–*10*).

PcG proteins are brought to their target genes by specific DNA fragments, Polycomb response elements (PREs) in *Drosophila* (*11*, *12*). In mammals, unmethylated CpG islands (CGIs) are often bound by PRC1 and PRC2, although these complexes appear to be recruited by different DNA sequences (*13*, *14*). PREs were identified in transgenes by their ability to recruit PcG proteins, act as repressive elements, and render transgene expression susceptible to mutations in PcG genes (*11*). Similarly, CGIs can bind PcG proteins and initiate the formation of H3K27me3 domains in mammals (*15*). CGIs are also important for gene activation as they serve as promoters for many developmental genes (*16*) as well as facilitate enhancer activity (*17*). PREs can also play a role in gene activation through mechanisms that are not well understood (*18*) but include their binding of activator proteins including trithorax group proteins (*19*, *20*).

PREs also interact with each other and influence three-dimensional (3D) chromatin structure (*21*, *22*). Hi-C and related techniques enable the detection of interactions of distal DNA sequences, which appear as loop-dots in the contact matrices. These loops are not stable structures, and looping may occur in only a fraction of cells at any one time (*23*, *24*). On the basis of Hi-C data, PREs form some of the strongest loops in *Drosophila* embryos (*21*). Recent micro-C data have achieved higher-resolution 3D chromatin maps and identified DNA anchors that mediate promoter-promoter and promoter-enhancer interactions which have been called “promoter-tethering elements” or PTEs (*25*, *26*). Many of these PTEs colocalize with PREs. A good example of how a PRE can function as a tethering element is found at the *engrailed* (*en*) locus which together with *invected* (*inv*) are within a canonical PcG targeted domain (i.e., the *inv-en* domain). A PRE at the *en* gene was called a “promoter-tethering element” because it facilitated the action of *en* imaginal disc enhancers on a transgene inserted near the *en* promoter (*27*).

Most studies on canonical PcG target genes in *Drosophila* have been done in either cell lines where these developmental genes are mostly silenced or in an “OFF” transcriptional state (hereafter abbreviated as OFF state) or in mixed cell populations of heterogeneous chromatin activity. Thus, we know a lot about the distribution of H3K27me3 and what proteins bind to PREs within genomic regions of the OFF state. PcG-repressed genes are covered by H3K27me3, and their PREs bind all PcG proteins tested. Less is known about PcG binding to PcG target genes in the ON state simply because these genes are OFF in most cell lines. However, we do know that PcG binding to some PREs occurs in the ON state, but the amount of binding is variable. Almost two decades ago, Schwartz *et al*. (*28*) found that the binding of Psc, Pc, and E(z) on the PREs near the actively transcribed *Abd-b* gene is much lower than those on PREs associated with the weakly transcribed *Ubx* gene. In the same year, Papp *et al.* compared PcG binding to the *Ubx* gene in two different imaginal discs, one that had the *Ubx* gene transcribed (ON) and the other OFF. They found that multiple PcG proteins (i.e., PhoRC, PRC1, and PRC2 components) remain bound to the *Ubx* PREs even in the ON state but often with reduced levels. Similar conclusions were reached in other studies with more highly transcribed genes likely to have lower levels of PcG binding (*19*, *29*, *30*). We also know that transcription alone does not remove PcG proteins from a PRE (*31*). Despite these important studies, examining the binding of PcG proteins to PREs in more PcG target genes in the ON state is important to know whether these results can be generalized.

Here, we examined what PcG proteins bind to PREs in the ON state and how chromatin looping changes between the ON and OFF states. PREs are a diverse group made up of binding sites for many different DNA binding proteins (*29*–*34*). We have been studying the four architecturally and functionally diverse PREs of *inv-en* for many years (*12*, *35*–*38*), focusing on their activity and PcG binding in the OFF state. Apart from PcG binding, a recent study showed that these PREs loop in embryos, a mixed cell population (*25*). We wondered what PcG proteins bind to these diverse PREs in cells that express *inv* and *en*. To examine binding in a single-cell type, we leveraged the modENCODE data to identify a ML-DmD17-c3 cell line with active *inv* and *en* expression and performed multiomic profiling of it and S2 cells to address three questions: (i) Are PcG proteins bound to the *inv-en* PREs in the ON state? (ii) Do PREs loop in the ON and OFF states? (iii) What happens to PcG binding and looping to PREs in other expressed canonical PcG target genes? Our results show that PcG binding to PREs in the ON state depends on the PRE, that PREs can loop with other PREs in both the ON and OFF states, and that PREs can also loop with presumptive enhancers.

## RESULTS

### Loss of H3K27me3 at three of the four canonical *inv-en* PREs in D17 cells

**Fig. 1.**
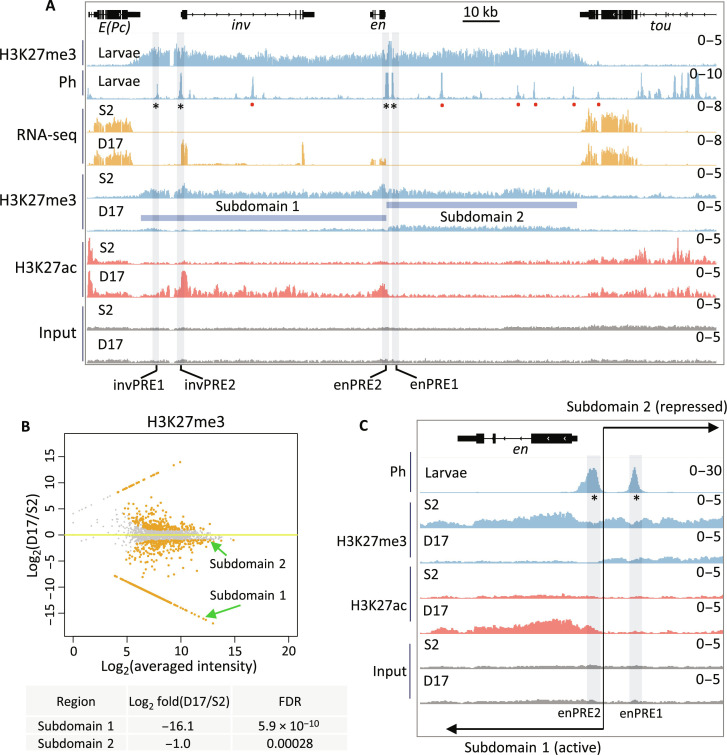
Transcription, PcG binding, and epigenetic patterns at the *inv-en* locus in S2 and D17 cells. (**A**) IGV tracks show the RNA sequencing (RNA-seq) and ChIP-seq data at the *inv-en* locus. The top two tracks show the H3K27me3 and Ph occupancy from larval brains and discs. The asterisks under the Ph peaks indicate the four major PREs, and the red dots represent minor PREs. The data for S2 and D17 cells are shown at bottom. The blue bars denote the subdomain 1 (H3K27ac covered) and subdomain 2 (H3K27me3 covered). (**B**) MA plot shows the alterations of H3K27me3 levels between D17 and S2 cells. The two subdomains of *inv-en* locus are highlighted by green arrows, with their detailed statistics shown at bottom. (**C**) Enlarged view of H3K27me3 and H3K27ac intensity over the two enPREs (asterisks). The two PREs fall into different subdomains with a precise border flanking enPRE2. The transition between the two subdomains is marked by the black line and opposing arrows.

The *inv-en* locus represents one of the most well-characterized PcG-targeted domains. In the OFF state, H3K27me3 spreads over the entire *inv-en* domain extending 113 kb from the 3′ end of *E*(*Pc*) to the 3′ end of *tou* (Fig. 1A). This is true not only in most cell lines (e.g., S2 cells used here) but also in mixed cell populations from larval discs and brains where 80% of the cells are estimated to be in the OFF state (*39*). Within the *inv-en* domain, there are four major (or constitutive) PREs, including two associated with *inv*, invPRE1 and invPRE2, and two upstream of *en*, enPRE1 and enPRE2 (Fig. 1A). These PREs can act in transgenes to silence gene expression (*36*, *37*) and are bound by PcG proteins in embryos, larvae, and all cell lines assayed to date. In larvae, there are a number of smaller peaks of PcG binding, so-called minor PREs (*40*), which are tissue specific and may be dual-functional elements that can act as either PREs or enhancers depending on the cell type (*41*). On the basis of chromatin immunoprecipitation sequencing (ChIP-seq) data, PcG binding to “minor” PREs is missing from cell lines but present in embryos (*40*).

All PcG proteins assayed bind the *inv-en* PREs in the OFF state, but what binds to them when *inv-en* are ON? We took advantage of the transcriptomic data generated by ModENCODE on 25 cell lines and found that ML-DmD17-c3 (hereafter called D17) is the only cell line that expresses *inv-en* at moderate levels (*42*). Then, we compared the *inv-en* expression in D17 cells to S2 cells, which do not express *inv-en*. Because cell lines can vary based on source, culture condition, and passage, we characterized the transcriptomic profiles for D17 and S2 cells grown in our laboratory (data S1). As expected, and crucial for this study, S2 cells express neither *inv* nor *en*, while D17 cells express both, with *inv* about threefold higher than *en* (Fig. 1A). Like in larvae, H3K27me3 covers the entire *inv-en* domain in S2 cells which indicates the OFF state. In contrast, the *inv-en* domain is broken into two subdomains in D17 cells, with subdomain 1 covered by H3K27ac starting upstream of *inv* through the *en* transcription unit, and subdomain 2 covered by H3K27me3 starting 600 bp upstream of the *en* transcription unit extending 50 kb to the *tou* gene (Fig. 1A).

Given that H3K27me3 covers all canonical PcG-repressed regions, we next compared its distribution between S2 and D17 cells by using ChIP-seq. In total, 434 genomic loci have significantly decreased H3K27me3 levels in D17 cells (data S2), and among them, *inv-en* subdomain 1 is ranked as the third most significant (Fig. 1B and data S2). Of note, the *Grip/mab-21* and *bab1/bab2* loci, two other PcG targets, are ranked as the top two. In contrast, subdomain 2 shows only slightly lower H3K27me3 level in D17 cells and ranked as 302 (Fig. 1B and data S2). These data show that about 50 kb of *en* regulatory DNA located in subdomain 2, including enhancers for stripes, nervous system, imaginal discs, etc. (*43*), is covered by H3K27me3 in D17 cells at approximately the same level as seen in cells in the OFF state. The two En PREs are in two different subdomains: enPRE2 in the H3K27ac domain and enPRE1 in the H3K27me3 domain (Fig. 1C). The transition between these two marks is abrupt and coincident with the rightmost end of PRE2. We also assayed four other active marks including H3K4me2/3 and H3K36me2/3 (fig. S1). As expected for active transcription, H3K4me3 covered the promoters of both *inv* and *en* in D17 but not S2 cells (fig. S1).

### PcG binding to PREs is altered in the ON state

**Fig. 2.**
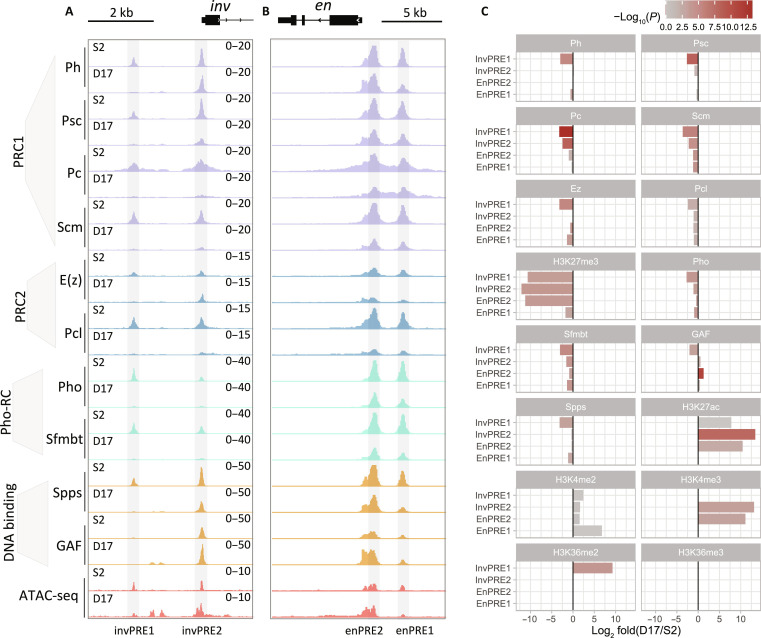
Binding of core PcG and related proteins at *inv-en* PREs in S2 and D17 cells. (**A** and **B**) IGV tracks show the occupancy of different PcG proteins from different complexes (PRC1, PRC2, and Pho-RC), DNA binding factors, and chromatin accessibility on the four major *inv-en* PREs in S2 and D17 cells. (**C**) Bar plots show the differences in ChIP signals for different PcG proteins and related factors at each individual *inv-en* PREs between D17 and S2 cells.

We next compared the PcG binding on PREs in the ON and OFF states, first focusing on the *inv-en* PREs that show altered chromatin states between S2 and D17 cells. For this purpose, we used ChIP-seq to determine the distribution of the following: (i) core PRC1 components (Ph, Psc, and Pc) and PRC1-associated protein (Scm); (ii) histone methyltransferase of PRC2 [E(z)] and the PRC2-associated component (Pcl); (iii) Pho-RC components (Pho and Sfmbt); (iv) PRE-DNA binding proteins [Spps and GAGA factor (GAF)]. Of note, Scm is associated with both PRC1 and PRC2 and plays a key role in their recruitment (*44*). The binding of many PcG proteins is greatly reduced to the Inv PREs and, less so, to the En PREs in D17 cells (Fig. 2 and fig. S2). Most notably, there is little to no binding of PcG proteins to invPRE1, located about 6 kb upstream of the *inv* transcription start site (TSS), and assay for transposase-accessible chromatin with high-throughput sequencing (ATAC-seq) data, a measure of open chromatin shows very little signal at this site in D17 cells (Fig. 2A). There is also weak or no binding of most PcG proteins to invPRE2, except for E(z), Ph, GAF, and Spps that do not differ much between S2 and D17 cells (Fig. 2A). Notably, there is very little GAF binding to invPRE1 in both cell types (Fig. 2A). Two additional GAF peaks are present near invPRE1 in D17 cells (Fig. 2A), and ATAC-seq shows that these peaks are correlated with open chromatin in D17 cells. At the *en* PREs, the differences in PcG protein binding in the ON and OFF states are less notable, with the binding of most PcG proteins retained with only a moderate reduction. This is consistent with our previous study that showed PcG protein binding to enPREs in larval brains and discs in ON cells (we did not assay the *inv* PREs) (*45*). Thus, although enPRE1 is covered by H3K27me3 and enPRE2 is covered by H3K27ac, this does not seem to affect the binding of most PcG proteins in these cell lines.

**Fig. 3.**
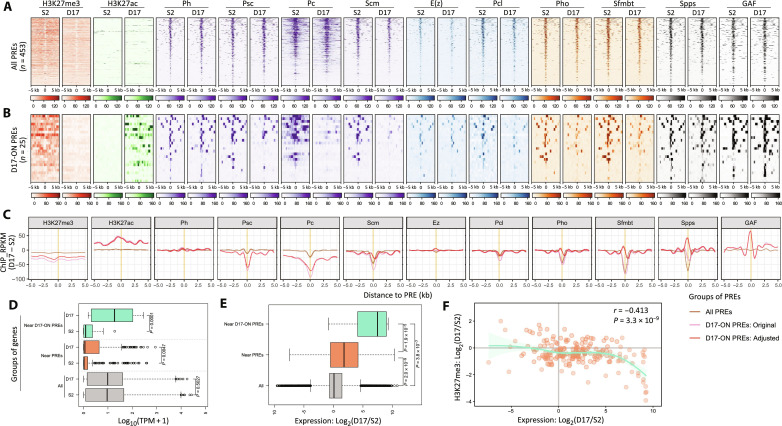
Altered binding of PcG-related proteins on PREs in S2 and D17 cells. (**A**) Heatmaps show the ChIP signal on all PREs. (**B**) Heatmaps show the ChIP signal on the D17-ON PREs. For the heatmaps in (A) and (B), the color gradients represent the reads per kilobase per million mapped reads (RPKM) values calculated from ChIP-seq data. (**C**) Differences of ChIP signal (D17 versus S2) on all PREs (brown) or D17-ON PREs (pink for the “original” difference and red for the “adjusted” difference). ChIP signal is normalized as RPKM values from the ChIP-seq data. The curves are smoothed by the “loess” method. (**D**) The expression level of different groups of genes in S2 and D17 cells. The gene groups include all genes (gray) or the genes with their TSSs less than 1 kb from any PREs (orange) or D17-ON PREs (green). Gene expression is evaluated as transcripts per million (TPM) values. The *P* values were calculated using two-sided Student’s *t* test. (**E**) Differential expression (D17 versus S2) for different groups of genes. The *P* values were calculated by two-sided Student’s *t* test. (**F**) The correlation between the altered expression and the altered H3K27me3 levels for PRE-adjacent genes (i.e., genes with their TSSs ≤1 kb to PREs). The Pearson’s *r* and *P* value are indicated.

As a control, we examined the Abd-B domain, which is silenced by PcG proteins in both S2 and D17 cells. While PcG binding to most PREs in this region looks qualitatively similar in the two cell types (fig. S3), the two PREs within the Abd-B transcription unit showed lower PcG binding in D17 cells (fig. S4). To achieve a global picture, we defined a set of all PREs (*n* = 453) as loci co-occupied by E(z), Ph, Pc, and H3K27me3 in either cell lines. The global levels of H3K27me3 as well as Psc, Pc, Scm, Pcl, Pho, Sfmbt, and Spps on them were lower in D17 cells (Fig. 3A). We further examined the D17-ON PREs (*n* = 25), which are OFF (H3K27me3^+^ and H3K27ac^−^) in S2 cells and ON (H3K27me3^−^ and H3K27ac^+^) in D17 cells, and found that the decrease of PcG binding on them is more evident than the trend for all PREs (Fig. 3B). Consistently, even after subtracting the overall alterations estimated from all PREs, the D17-ON PREs still showed decreased binding of most PcG proteins (with the exceptions of Ph, Spps, and GAF) in D17 cells (Fig. 3C), confirming the influence of transcriptional state on the PcG binding on PREs. We also compared the expression level of genes adjacent to different groups of PREs. We confirmed the relatively low expression of PRE-adjacent genes and the increased expression of genes adjacent to D17-ON PREs in D17 cells (Fig. 3, D and E). Significantly higher expression was observed for genes adjacent to all PREs (instead of only D17-ON PREs) in D17 relative to S2 cells (Fig. 3, D and E), probably due to the lower H3K27me3 levels in D17 cells since lower H3K27me3 levels correlate well with increased expression levels of PRE-adjacent genes (Fig. 3F).

### Putative enhancers underlie the D17-specific *inv-en* expression

**Fig. 4.**
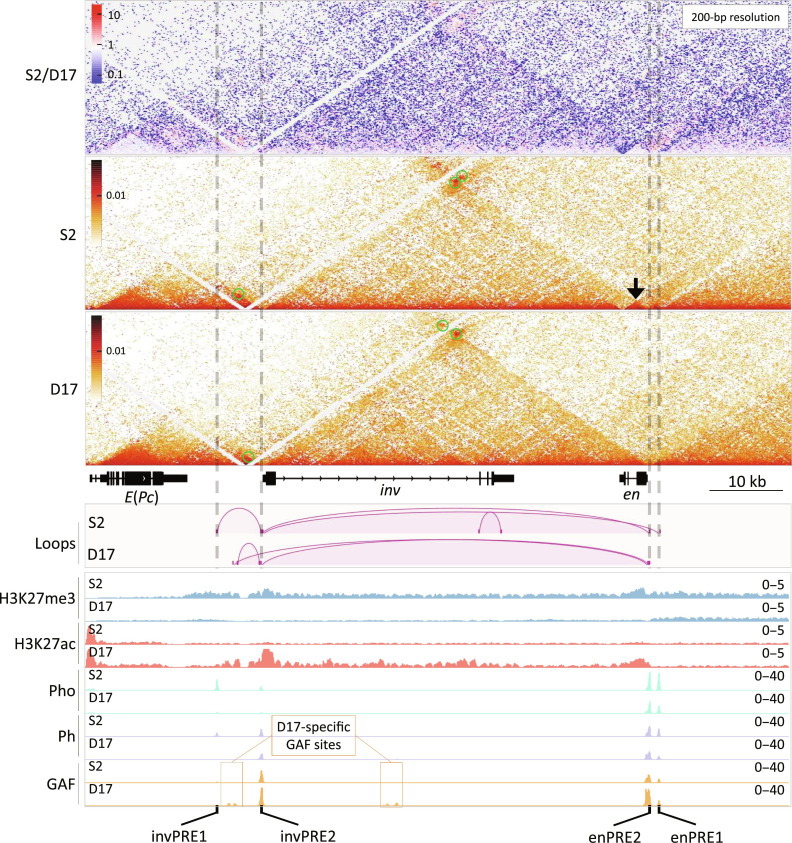
PcG binding and chromatin structure over the *inv-en* domain in S2 and D17 cells. The contact map at top shows the differences between S2 and D17 cells, while the other two are for each cell type. The arrow points to the *en* transcription unit that forms its own small domain in S2 cells, whereas it is in the same domain as *inv* in D17 cells. The significant loops are visualized as arcs below the gene models, and the corresponding loop-dots are also highlighted by green circles in the contact maps. ChIP-seq tracks for H3K27me3, H3K27ac, Pho, GAF, and Ph are shown at the bottom, with the D17-specific GAF binding sites highlighted in orange rectangles. The positions of the *inv-en* PREs are indicated by dashed lines at the top and labeled at bottom.

What regulatory regions are driving *inv-en* expression in D17 cells? Previous studies on the *Ubx* gene in *Drosophila* and HOX genes in mice suggest that active enhancers are enriched in H3K27ac (*46*, *47*), thus we reasoned that the enhancers that stimulate *inv-en* expression should also be marked with H3K27ac. In addition, enhancers should be accessible and may regulate their target genes through enhancer-promoter contact (*48*, *49*). Accordingly, we screened for the enhancers that may drive *inv-en* expression in D17 cells based on three criteria: (i) ChIP-seq to identify H3K27ac peaks; (ii) ATAC-seq to identify accessible chromatin regions; (iii) micro-C to identify regions that contact the *inv-en* promoters (Fig. 4). In S2 cells, ATAC-seq shows enriched accessible chromatin in the *inv-en* domain only at the four PREs (Fig. 2 and S5). PREs bind many different proteins, are nucleosome-depleted regions, and are detected as “accessible” chromatin (*50*, *51*). In D17 cells, there are additional regions of chromatin accessibility, within the *inv* intron and upstream of the *inv* promoter (Fig. 2 and fig. S5). These regions also correlate with cell type–specific GAF binding sites and weak peaks of Spps and Ph (fig. S5). In the intron of *inv*, these new GAF peaks are contained within a fragment of DNA that can act as an enhancer in wing imaginal discs (*52*). One of the new GAF peaks coincides with a larval Ph peak and may represent a dual function element that serves as an enhancer in some cell types and PRE in others (*41*). We suggest that this enhancer may contribute to *inv* and *en* expression in D17 cells.

### PREs loop in the ON state

We performed micro-C to compare the chromatin organization in the *inv-en* region between S2 and D17 cells. The two genes *inv* and *en* reside in a large domain in both cell types, but the subdomains differ (Fig. 4 and fig. S6). Specifically, the *en* transcription unit itself forms its own small subdomain in S2 cells (arrow) yet is contained within the same subdomain as *inv* in D17 cells. PREs are known to promote strong focal interactions that can be described as loops (*21*). Consistent with this, PRE-mediated interactions are evident based on the contact maps and loop calling results (Fig. 4 and data S3). In S2 cells, significant loops form between the two *en* PREs and invPRE2 and also between the two *inv* PREs, with the loop between the two promoter-associated PREs (invPRE2 and enPRE2) the strongest. Despite the fact that GAF can mediate the looping of some tethering elements (*53*), invPRE1 has very weak GAF binding yet still interacts with invPRE2 in S2 cells (Fig. 4), indicating that their interaction is probably GAF independent. In contrast, very low levels of PcG proteins and almost no GAF are bound to invPRE1 in D17 cells, and invPRE1 does not form any loops in D17 cells (Figs. 2A and 4). Instead, a new interaction arises upstream of the *inv* promoter, near the position of two D17-specific GAF binding sites (Figs. 2A and 4). While it is tempting to speculate that invPRE2 can either interact with invPRE1 or the new GAF peaks, the simplest interpretation of our data is that these two PREs cannot form loops when they are not bound by PcG proteins. invPRE1 can function on its own as a PRE in transgenes (*36*). Also, although not detected as significant loops, there are two dots in the exact locations of invPRE1-enPRE2 and invPRE1-enPRE1 in S2 cells, suggesting that invPRE1 may be able to weakly interact with the enPREs. Overall, our data show that when PREs are bound by at least a subset of PcG proteins, the PRE–PRE promoter–proximal loops are present in both the ON and OFF states. We hypothesize that enPRE1 does not loop with invPRE2 and enPRE1 in D17 cells because it is covered with H3K27me3 instead of H3K27ac.

### PcG protein binding and chromatin looping of PREs in other canonical PcG targets

Given that thousands of genes are transcribed differentially in S2 and D17 cells (data S4 and fig. S7), we performed a genome-wide search for all PcG target genes that are (i) exclusively transcribed in one cell type as judged by RNA sequencing (RNA-seq), (ii) covered with H3K27me3 in the OFF state and H3K27ac in the ON state, and (iii) have PcG peaks indicative of PREs within the H3K27me3 domain. After manual inspection of each candidate locus, we identified four additional PcG domains that had gene(s) ON in D17 cells and OFF in S2 cells, including *bab1-bab2*, *mab-21*, *croc*, and *zfh2*. In contrast, no genes that met these criteria were ON in S2 cells and OFF in D17 cells. Therefore, very few genes were identified as canonical PcG targets despite the thousands of genes showing altered expression between S2 and D17 cells (data S4 and fig. S7). Subsequently, we inspected the Polycomb protein binding and chromatin organization on these four canonical PcG domains.

**Fig. 5.**
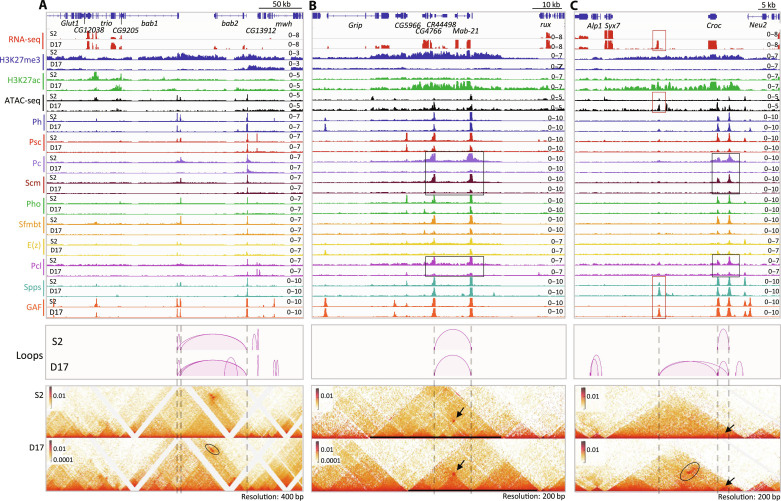
Transcription, PcG binding, and chromatin structure over the *bab1/bab2*, *mab-21*, and *croc* domains in S2 and D17 cells. The top are for the RNA-seq, ATAC-seq, and ChIP-seq data, and the bottom are for micro-C data. The significant loops are visualized as arcs at the top of the contact maps. The dashed lines highlight the presumptive PREs. (**A**) *bab1/bab2*. The oval in the contact maps highlight an area of different chromatin structure between S2 and D17 cells. (**B**) *mab-21*. The rectangles highlight the major differences in the binding of the PRC1 components Pc and Scm, and the PRC2 associated protein Pcl between S2 and D17 cells. The arrows points to the interaction of the two major PREs that is present in both cell types. At this locus, the changes of the domains do not correlate with changes in PRE binding. (**C**) The black rectangles highlight the altered binding of Pc, Scm, and Pcl between S2 and D17 cells. The red rectangles highlight a region with elevated binding of GAF, Spps, and chromatin accessibility and a previously unknown transcript in D17 cells. The oval in the contact map highlights the interaction of the *croc* PREs with this accessible region in D17 cells. The arrows point to the interaction between the two PREs upstream of *croc*.

The *bab1-bab2* domain has three PREs: two at *bab1* and one at *bab2* (Fig. 5A). In S2 cells, the H3K27me3 domain starts ~200 kb upstream of the 3′ end of *bab1* and extends over *bab2* and several other nontranscribed genes until it reaches *Klp61F* that is transcribed in both cell types (Fig. 5A and fig. S8). The H3K27me3 domain is smaller in D17 cells; it begins just upstream of the *bab2* PRE. In D17 cells, most PcG proteins, except for E(z), Ph, and GAF, are almost completely depleted from the *bab1* PREs (Fig. 5A), and H3K27ac is increased over the *bab2* transcription unit and the 5′ end of the *bab1* promoter (fig. S8). While the *bab2* PRE loops with both *bab1* PREs in both cell types, we observed a D17-specific stripe (oval) (Fig. 5A). We hypothesize that this stripe is formed by the process of loop extrusion mediated by the cohesin complex, as described in the review by Davidson and Peters (*54*).

The *mab-21* domain has two PREs: one over the *mab-21* promoter and the other one over the *CR44498/CG4766* promoters. H3K27me3 spreads over a 43-kb region including *Grip* (3′end), *CG5966*, *CG4766*, *CR44498*, and *mab-21* in S2 cells but is lost from the whole region in D17 cells (Fig. 5B). In contrast, H3K27ac covers the 3′end of *CG5966* to the *mab-21* promoter in D17 cells, and multiple genes including *Grip*, *mab-21*, *CR44498*, and *CG4766* show D17-specific expression. In D17 cells, the levels of Pho, Psc, Sfmbt, and E(z) are reduced at both PREs, with the biggest reductions observed for Pc, Pcl, and Scm. In contrast, the levels of GAF, Spps, and Ph remain largely unchanged. The chromatin structure differs substantially in the two cell types: A large domain enriched with H3K27me3 is seen in S2 cells, which is heavily reconfigured at both sides in D17 cells. Notably, despite the strong differences in domain structure the two PREs form the same loop in both the ON and OFF states (Fig. 5B, arrow). However, unlike at *inv-en*, we did not detect any new PRE-mediated interactions in the ON state.

The *croc* and *zfh2* domains have three and five PREs, respectively. Similar to the *mab-21* domain, H3K27me3 is lost from both domains in D17 cells, and H3K27ac occupies (part of) the same region (Fig. 5C and fig. S9). Also, the binding of most PcG proteins is decreased in D17 cells, with the exception of Ph, GAF, and sometimes Spps. The loops between the PREs are still present in the ON state but become weaker in the *zfh2* domain (Fig. 5C and fig. S9). D17-specific binding of GAF and Spps appears ~13 kb downstream of the *croc t*ranscription unit, which corresponds to an unannotated 400-bp transcript in D17 cells (Fig. 5C). We suggest that this transcript may be the rare example of a stable enhancer RNA in *Drosophila*. This region also has D17-specific interactions with the two PREs (Fig. 5C, oval); thus, it could represent a D17-specific enhancer. In sum, the ability of PREs to bind some PcG proteins and mediate specific strong focal looping interactions in the ON state can be observed at multiple genetic loci.

## DISCUSSION

Polycomb repression of developmental genes is key to proper development of an organism. In the OFF state, PcG proteins bind PREs and H3K27me3 covers entire genes/gene complexes. Polycomb also plays a role in genome organization. Distant H3K27me3 domains colocalize in both *Drosophila* and mammalian genomes (*55*, *56*), and PREs form loops to stabilize gene repression in *Drosophila* (*21*). PREs can also play a role in gene activation and can transmit a memory of both the ON and OFF states (*19*). In this regard, PREs can also be bound by trithorax group proteins, such as Fs(1)h and Trx (*57*). In addition, Fs(1)h and the coactivators Enok/Br140 copurify with the PRC1 proteins Pc and Psc (*58*, *59*). Last, recent biochemical studies have shown that Sfmbt interacts with another group of coactivators that are homologous to coactivators linked to the mammalian YY1-MBTD1 complex, the mammalian counterpart of PhoRC (*44*). These studies all suggest that PREs can function in either gene activation or gene repression.

What PcG proteins bind to PREs in a canonical PcG-targeted developmental gene that is ON? Most previous studies in cell lines, embryos, and imaginal discs showed that PcG proteins remain bound to PREs of actively transcribed genes but at reduced levels (*20*, *46*). Aside from a groundbreaking study on two *Ubx* PREs in imaginal cells (*20*), most studies examined the binding of only a few PcG proteins. We examined the distribution of the following: (i) the PRC2-associated repressive histone mark H3K27me3; (ii) the active histone mark H3K27ac; (iii) Ph, Psc, and Ph from canonical PRC1; (iv) E(z), the histone methyltransferase in PRC2, Pcl, a component of one form of PRC2 that is important for achieving high levels of H3K27me3, Scm, which is associated with both PRC1 and PRC2; (v) the PhoRC components Pho and Sfmbt; (vi) the PRE DNA binding proteins Spps and GAF. The major conclusions that can be made from our work are as follows: (i) Like the Hox genes in both *Drosophila* and mammals, inactive regulatory DNA can be covered with H3K27me3, while transcription units and active regulatory DNA are covered by H3K27ac. (ii) In the ON state, the binding of many PcG proteins is decreased on some PREs while almost completely lost on other PREs within the same gene/gene complex. (iii) Ph, GAF, and Spps remain at most PREs even when other PcG proteins are greatly reduced, and particularly, Ph seems to stay at the PREs in the absence of other PRC1 components (i.e., Pc, Psc, Scm)—suggesting that Ph binds either by itself or in another protein complex. (iv) E(z) is at most PREs even when they are within H3K27ac domains, while Pcl frequently shows reduced binding when a PRE is turned ON, consistent with the differential inclusion of Pcl in different forms of PRC2 complexes (*6*). (v) For PREs with markedly reduced binding of many PcG proteins, they can still loop if Ph and GAF binding are maintained. (vi) PREs loop with each other in the ON state but can also loop with other elements (i.e., presumed active enhancers) in developmental loci that are being expressed.

Chromatin marks and PcG binding to *inv-en* have been studied in BG3 cells (a cell line derived from larval brain and ventral ganglion) and in imaginal disc cells that express *inv-en*. In BG3 cells, where *inv-en* are expressed at low levels, H3K27me3 covers the entire *inv-en* domain (*19*, *60*), and Hi-C data showed a loop between the *inv* and *en* promoter regions (*61*). The resolution of the Hi-C data is not high enough to distinguish the individual PREs. Pc is bound to the *inv-en* PREs in BG3 cells, but unlike in cells where *inv-en* are OFF, the activator Ash1 is bound in the vicinity of the *inv-en* promoters (*19*). Cohesin is bound to *inv-en* promoters and intervening DNA in BG3 cells and reducing the amount of cohesin or Polycomb proteins by RNAi caused an increase in *inv* and *en* expression (*62*). Therefore, the authors posited that cohesin and PcG complexes interact to constrain *inv-en* expression in this cell line. Here, we did not look at the binding of Ash1 or cohesin since we failed to obtain Ash1 antibody and good ChIP-seq data for a cohesin component. Nevertheless, it is clear that the chromatin of the *inv-en* locus in BG3 cells is quite different from D17 cells.

In another study, wing imaginal discs were dissected into anterior (*inv-en* OFF) and posterior compartments (*inv-en* ON), and then H3K27me3 levels were assessed by ChIP-seq (*60*). As expected, high levels of H3K27me3 covered the entire *inv-en* domain in cells of the anterior compartment. The posterior cells were covered by a low level of H3K27me3, with significant peaks absent in the 50 kb of regulatory DNA upstream of the *en* PREs where the enhancers for expression in the posterior compartment are located (*63*). The investigators noted that dissecting the discs into posterior and anterior compartments was difficult, and they estimated that the posterior cells could be contaminated with about 10% anterior cells. We suggest that the lack of significant H3K27me3 upstream of *en* could indicate that this regulatory region is not trimethylated in cells that express En, similar to what we saw in D17 cells.

Our study supports the view that PREs are one class of PTEs in the *Drosophila* genome. In the OFF state, PREs loop to each other and strengthen PcG-mediated gene repression (*21*). Here, we show that in the ON state, PREs can also loop to each other and to presumptive enhancers. The DNA binding proteins GAF, Spps, and the PcG protein Ph are retained at high levels at most PREs in the ON state. In agreement with a previous report that GAF fosters loop formation in *Drosophila* genome (*53*), our data suggest that GAF may contribute to looping of PREs with enhancers. However, we note that mutating GAF did not cause a loss of most PTE loops (*53*). Could Ph be playing a role in looping? Mutating GAF binding sites in enPREs 1 and 2 abrogated GAF binding but only reduced Ph binding about 50% (*38*). We suggest that, like for PcG recruitment at PREs, looping may be mediated by a combination of proteins with overlapping activities.

While this study comprehensively profiled the binding of various PcG proteins on PREs, we did not examine many other proteins known to bind PREs, such as Fs(1)h and Trx which belong to trithorax group proteins (*19*, *57*) and Enok which is an activator protein (*58*). As stated above, Sfmbt interacts with another group of coactivators that are homologous to coactivators linked to the mammalian YY1-MBTD1 complex, the mammalian counterpart of PhoRC (*44*). These proteins might also bind PREs in a differential manner. Thus, we cannot say whether the same proteins mediate PRE looping in the ON and OFF transcriptional states. Despite this limitation, our study is an important first step in addressing this fundamental question.

## MATERIALS AND METHODS

### Cell culture

S2-DRSC and ML-DmD17-c3 cells came from the Drosophila Genomics Resource Center (DGRC), with stocks 181 and 107, respectively (https://dgrc.bio.indiana.edu). Both cell lines were grown on M3 + BPYE + 10% heat-inactivated fetal bovine serum (Hyclone SH30070.02) as recommended by DGRC (https://dgrc.bio.indiana.edu/include/file/TissueCultureMedium.pdf). The medium for D17-c3 cells were supplemented with insulin (10 μg/ml). Both cell lines were grown at 25°C.

### ChIP-seq

Detailed experimental procedure for ChIP-seq are provided in Supplementary Materials and Methods. The antibodies used are summarized in data S5. The raw reads were first trimmed with TrimGalore v0.6.4 and then aligned to the *Drosophila* reference genome (BDGP6) using Bowtie v2.3.5.1 (*64*) with settings: --local --very-sensitive-local --no-unal --no-mixed --no-discordant -I 10 -X 1000. Polymerase chain reaction duplicates were removed using the *rmdup* function of samtools v1.13 (*65*). After confirming the data reproducibility, the replicates were pooled together for further analysis. Peak calling was performed using MACS v2.2.6 (*66*) with settings: -f BAMPE --keep-dup all --fe-cutoff 1.5 -q 0.05 -g dm. The peaks were further filtered by removing those that overlap ENCODE Blacklist V2 regions (*67*). Differential binding sites were determined using DiffBind v3.0.15 (*68*) with settings: minOverlap = 0, summits = 250, method = DBA_EDGER, false discovery rate (FDR) < 0.05, |log_2_ fold change| > 1. Notably, for H3K27me3 domains which are made up of multiple subdomains that change differently (e.g., the H3K27me3 domain flanking *inv* and *en* genes), we manually split the entire domain as subdomains based on the peak calling in the S2 and D17 cells, which were then subjected to differential binding analysis.

### ATAC-seq

ATAC-seq and library preparation were performed using the Active Motif ATAC-seq kit (#53150) following the manufacturer’s instruction. ATAC-seq data were analyzed following the same procedure as for ChIP-seq data.

### RNA sequencing

Detailed experimental procedure for RNA-seq are provided in Supplementary Materials and Methods. Raw reads were trimmed with Trim Galore v0.6.4. and then aligned to the *Drosophila* reference genome (BDGP6) using STAR v2.7.3a (*69*). Gene-level read counts were calculated by using the *featureCount* function from subread v2.0.0 (*70*). Differentially expressed genes were identified using DESeq2 v1.30.1 (*71*) with the cutoff: FDR < 0.05, |log_2_ fold change| > 1. TPM values were calculated by using RSEM v1.3.2 (*72*).

### Micro-C

Micro-C experiments were performed as described previously (*73*) with a few modifications. Following binding of micro-C fragments to streptavidin beads, libraries were prepared as described for Hi-C libraries in (*74*), with the detailed procedure provided in Supplementary Materials and Methods. Micro-C data were analyzed using HiC-Pro (*75*) without specifying a restriction enzyme site and filtering out interactions within 400 bp. The mcool files generated using HiC-Pro were then visualized in the resgen.io browser (https://resgen.io/pedrorocha/Kassis/views) (*76*, *77*). Significant chromatin loops were called using MUSTACHE v1.3.2 (*78*) with settings: -r 200 -st 0.8 -pt 0.05. Two independent pools of fixed cells from each cell line were analyzed separately, and duplicate reads were summed. Each of these pools was sequenced twice, and duplicated reads were removed.

### PRE annotation

We defined PREs as genomic loci co-occupied by Ph, Pc, E(z), and H3K27me3 signals as determined by our ChIP-seq peaks. By using the data for S2 and D17 cells, we defined 453 PREs in total. Among them, 25 PREs were further determined to be turned ON in D17 cells (denoted as D17-ON) based on the gaining of H3K27ac signal and loss of H3K27me3 signal in D17 relative to S2 cells.

### Reference genome and annotation

Reference genome and gene annotation for *Drosophila* (BDGP6) were downloaded from the ENSEMBL database (release 100) (*79*). The ENCODE Blacklist V2 regions for *Drosophila* were downloaded from ENCODE project (*67*).

### Statistical analysis and data visualization

All statistical analyses were performed with R statistical programming language (*80*). Heatmaps for ChIP-seq data were generated using DeepTools v3.5.1 (*81*). Heatmaps for gene expression clustering analysis were generated in R using pheatmap package. RNA-seq, ChIP-seq, and ATAC-seq tracks were visualized using Integrative Genomics Viewer v2.11.1 (*82*).

## Supplementary Materials

### This PDF file includes:

Supplementary Materials and Methods Figs. S1 to S9 Legends for data S1 to S5 References

### Other Supplementary Material for this manuscript includes the following:

Datas S1 to S5
