# Supplementary material for: Polycomb protein binding and looping in the ON transcriptional state
Source: Sci Adv. 2024 Apr 24;10(17):eadn1837. doi: 10.1126/sciadv.adn1837 (PMC11042752; doi:10.1126/sciadv.adn1837)
Supplement: Supplementary file 1 — Supplementary Materials and Methods Figs. S1 to S9 Legends for data S1 to S5 References [file sciadv.adn1837_sm.pdf]

Science Advances
AAAS

Supplementary Materials for

Polycomb protein binding and looping in the ON transcriptional state

J. Lesley Brown *et al.*

Corresponding author: Judith A. Kassis, jkassis@mail.nih.gov; Ming-an Sun, mingansun@yzu.edu.cn

Sci. Adv. **10**, eadn1837 (2024)
DOI: 10.1126/sciadv.adn1837

The PDF file includes:

Supplementary Materials and Methods
Figs. S1 to S9
Legends for data S1 to S5
References

Other Supplementary Materials for this manuscript include the following:

Data S1 to S5

Supplementary Materials and Methods

ChIP-seq

Cell fixation: For each cell type started with 30 mL of cells at 6×10^6 cells/ml. Added 1.87 mL of 16% formaldehyde and incubated at RT for 10 min with gentle rocking. The formaldehyde was quenched by adding glycine to a final concentration of 0.125 M followed by a 5 min incubation at RT with gentle rocking. The cells were spun down at 2000 g, 3 min, and washed twice with ice cold PBS. The cells were resuspended in 600 µL PBS, divided into 100 µL aliquots, spun down and the pellets were flash frozen and stored at -80 °C. Each aliquot of cells can be used for up to 5 ChIP reactions. Cells were fixed and frozen from different flasks to generate independent biological replicates.

X-ChIP: The frozen formaldehyde fixed cell pellets were resuspended in 0.8 ml ice-cold cell lysis buffer (5 mM PIPES pH 8, 85 mM KCl, 0.5% NP40, supplemented with protease inhibitors (Roche Complete EDTA-free protease inhibitor), incubated on ice for 10 min, then pelleted by centrifugation at 2000 g for 5 min at 4 °C. The supernatant was removed, and the pellet resuspended in 1 mL nuclear lysis buffer (50 mM Tris-HCl pH 8, 10 mM EDTA, 0.2% SDS, supplemented protease inhibitors) and incubated for 10 min at 4 °C on a rocking platform. 0.5 ml of 400 mM NaCl IP dilution buffer (16.7 mM Tris-HCl pH 8, 1.2 mM EDTA, 400 mM NaCl, 1.1% Triton X100, 0.01% SDS, supplemented with protease inhibitors) was added then gently mixed. The lysate was sonicated in 300 µL aliquots using a Q Sonica (Model Q800R3), at 40% amplitude for 30 sec OFF and 30 sec ON for a total of 5 min ON time. The sonicated samples were spun for 10 min at full speed in, an Eppendorf centrifuge at 4 °C. The 300 µL aliquots of each cell type sample were pooled again. 4 µL was removed as input (2% of a single ChIP reaction). To the remainder 100 µL of TE washed Protein A SepharoseTM Fast Flow (Cytiva) was added, and the samples were incubated at 4 °C for 1 hour with gentle rocking. The samples were spun at full speed in an Eppendorf centrifuge for 1 min, and the supernatant transferred to a fresh tube. For each IP, 200 µL of sonicated the sample was used + 800 µL 67 mM NaCl ChIP dilution buffer (16.7 mM Tris-HCl pH 8, 1.2 mM EDTA, 67 mM NaCl, 1.1% Triton X100, 0.01% SDS, supplemented with protease inhibitors. The appropriate amount of antibody (Supplemental Table 1) was added and incubated rocking overnight at 4 °C. 60 µL protein A/agarose bead slurry (prewashed with TE buffer prior to use) for 1 hour at 4 °C with rotation. The agarose was pelleted by centrifugation at 800 rpm at 4 °C for 1 min, the supernatant was carefully removed and the agarose was washed 5 min on a rotating platform at 4 °C sequentially with 1ml of the following buffers: Low salt immune complex buffer (0.1% SDS, 1% Triton X-100, 2 mM EDTA, 20 mM Tris-HCl, pH 8.0, 150 mM NaCl), High salt immune complex wash (0.1% SDS, 1% Triton X-100, 2 mM EDTA, 20 mM Tris-HCl pH 8.0, 200 mM NaCl), LiCl immune complex wash (0.25 M LiCl, 1% NP40, 1% deoxycholic acid (sodium salt), 1 mM EDTA, 10 mM Tris pH 8.0) followed by two 5 min washes with TE buffer. The agarose was pelleted again at 800 rpm for 1 min and the DNA was eluted with 500 µL freshly made elution buffer (1% SDS, 0.1 M $NaHCO_3$) for 30 min with gentle rocking. The agarose was pelleted, and the supernatant transferred to a fresh tube. The crosslinks were reversed by adding 20 µL of 5 M NaCl and incubating at 65 °C for 4 hours. The cross links were also reversed in the input sample. 10 µL of 0.5 M ETDA, 20 µL of 1 M Tris-HCl pH 6.5 and 2 µL of 10 mg/mL proteinase K were added and the samples incubated at 45 °C for 1 hour. The DNA was recovered by phenol/chloroform extraction followed by ethanol precipitation with 2 µL of pellet paint, 50 µL 5 M NaOAc, 1 mL Ethanol. Washed and dried pellets were resuspended in PCR grade water.

ChIP-seq library construction: 1.5 ng of ChIP sample DNA (as measured by a Qubit 3.0 fluorimeter) was used to prepare each library. Libraries were made using either the Thruplex DNA-seq and dual index kits (Takara), or the NEBNext Ultra^{TM} II DNA library preparation kit and dual index kits (New England Biolabs). Libraries were made according to the manufacturer's directions. Samples were sequenced by 50 bp pair-end sequencing with a NovaSeq 6000 with an SP100 kit by the NICHD Molecular Genomics Core.

RNA-seq

Library preparation and sequencing: For each cell line RNA was made from 4 separate tissue culture flasks to generate four independent replicates. For each RNA prep 1×10^7 cells spun down (1000 g, 3 min), washed twice with ice-cold PBS, and resuspended in 1 mL of ice-cold PBS, transferred to a 1.5 mL Eppendorf tube and spun down at full speed in a microfuge for 2 min at 4 °C. The pellet was resuspended in 100 µL PBS. Added 1 mL of Trizol to the cell suspension and vortexed for 1 min. Incubated the samples at RT for 15 min. Added 200 µL of chloroform and vortexed for 2 min. Centrifuged the samples at 13,200 rpm 15 min at 4 °C. Carefully transferred the top aqueous layer containing the RNA to a fresh tube. Measured the RNA concentration using the broad range Qubit RNA kit and a Qubit 3.0 fluorimeter (Invitrogen). An aliquot of the total RNA was further purified using the Qiagen RNeasy Micro kit following the manufacturer's directions. Libraries were made using the Illumina TruSeq Stranded mRNA sample prep kit, and then run on a NovaSeq 6000 using a SP 200 kit by the NICHD Molecular Genomics Core.

Micro-C

First, 25 million cells were pelleted and then fixed with DSG followed by formaldehyde (FA). 50 mg of DSG was resuspended in 500 µL DMSO and diluted with 50 mL PBS. The cells pellets were resuspended in the DSG solution at a concentration of 1×10^6 cells/mL. Cells were incubated gently rocking for 35 min at room temperature (RT). 16% FA was added dropwise to a final concentration of 1%, incubated 10 min rocking at RT. Glycine was added to a final concentration of 0.13 M, incubated 5 min at RT and 5 min on ice. Cells were pelleted at 1000 g for 5 min, washed with ice-cold PBS at a concentration of 1×10^6 cells/mL, centrifuged at 2500 g for 5 min at 4 °C, then washed with ice-cold PBS at a concentration of 1×10^6 cells/100 µL. Cells were counted the aliquoted in 1 or 5×10^6 aliquots in protein low bind tubes. Cells were centrifuged at 2500 g for 5 min at 4 °C, and the cell pellets, flash frozen -80 °C. MNase was titrated with 1×10^6 cells at 3U, 5U and 7U for 20 min at 37 °C. The DNA purification step was carried out using a Zymoclean DNA clean and concentrator kit. All DNA concentrations were measured with a Qubit and fragment sizes were assessed using a high sensitivity Tapestation (Agilent). The micrococcal nuclease reaction was carried out with 5×10^6 cells, and the micrococcal nuclease step was scaled up to 500 µL with the optimal concentration of micrococcal nuclease, incubation was at 37 °C was for 20 min. Centrifugation steps were at 3000 g. BSA to 100 µg/mL was added to MB#2 and MB#3 solutions just before use to help pellet disruption. DNA fragment end repair was carried out in a 95 µl end chewing Master mix + 5 µl 10U/µL PNK. 50µL of end labeling mix was added per sample. Centrifugations were at 5000 g. For proximity end ligation, pellets were resuspended in 500 µL of ligation master mix. After phenol/chloroform/ iso-amyl alcohol extraction, the sample was split into two equal aliquots and was purified on two Zymo DNA clean and concentrator kit columns. Samples were eluted with 25 µL (preheated to 70 °C) elution buffer then pooled. Samples were loaded onto the 3% TBE NuSieve GTG agarose gel in 4 separate wells. After excision from each lane samples were purified using a Zymo Gel DNA Recovery kit to extract the DNA. Elution was with 75 µL

elution buffer preheated to 70 °C. The 4 samples were then pooled to give a 300 µl sample. Enrichment of dinucleotides was confirmed by running an aliquot on an Agilent high sensitivity Tapestation. For biotin purification, 50 µL of Streptavidin beads were washed twice with 400 µL TBW, resuspended in 300 µL 2× BW then added to the 300 µL Micro-C sample followed by a 50 min incubation rotating at RT. Beads were washed twice with 600 µL TBW at 55 °C in a Thermomixer for 2 min. The beads were washed one time with 100 µL 10mM Tris, then resuspended in 50 µl 10 mM Tris. Libraries were prepared using the KAPA Biosystems HyperPrep kit and Illumina primers. End repair and A tailing was carried out as recommended by the manufacturer, adapter ligation was carried out with 1 µL annealed primer at 15 µM, ligation was 60 min at RT, gently mixing the beads every 10 min. 300 µL TWB was added, vortexed briefly and placed on a magnet and the supernatant removed. The beads were washed as above with 600 µL TWB, followed by 100 µL 10 mM Tris-HCl (pH8.0) then resuspended in 84 µL 10 mM Tris-HCl (pH8.0). After running a small-scale PCR to determine the optimal number of cycles for the required yield., 4×20 µL PCR reactions were set up for each sample. PCR: 98 °C 120 seconds, (98 °C 30 seconds, 65 °C 20 seconds, 72 °C 15 seconds) × 12 cycles, 72 °C 3 min. PCR reactions were pooled, the beads removed on a magnetic separator. 200 µL was transferred to a new tube and incubated with 0.9x SPRI beads. The final elution step is with 25 µL of 10 mM Tris-HCl (pH8.0). Samples were sequenced by 50 bp pair-end sequencing with a NovaSeq 6000 with an SP100 kit by the NICHD Molecular Genomics Core.

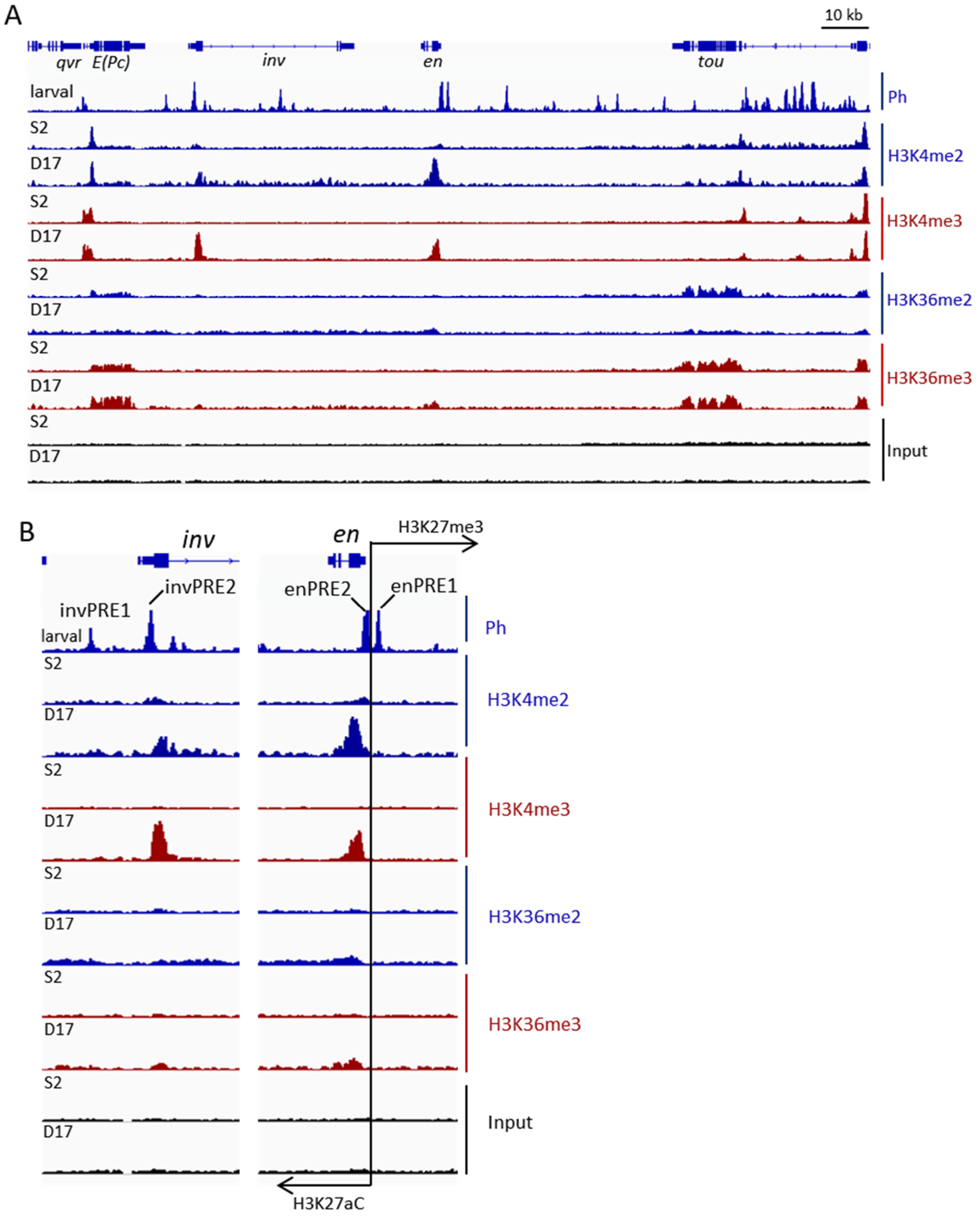

Fig. S1. Distribution of H3K4me2/3 and H3K36me2/3 over the *inv-en* locus in S2 and D17 cells

(**A**) IGV tracks show the distribution of H3K4me2, H3K4me3, H3K36me2 and H3K36me3 over the *inv-en* locus and the neighboring genes *E(Pc)*, and *tou*. A Ph track from larval tissue is included to indicate the positions of the PREs. All tracks are scaled at 0-7. (**B**) Similar to A, but shows an enlarged region for the *en* and *inv* PREs and transcription units.

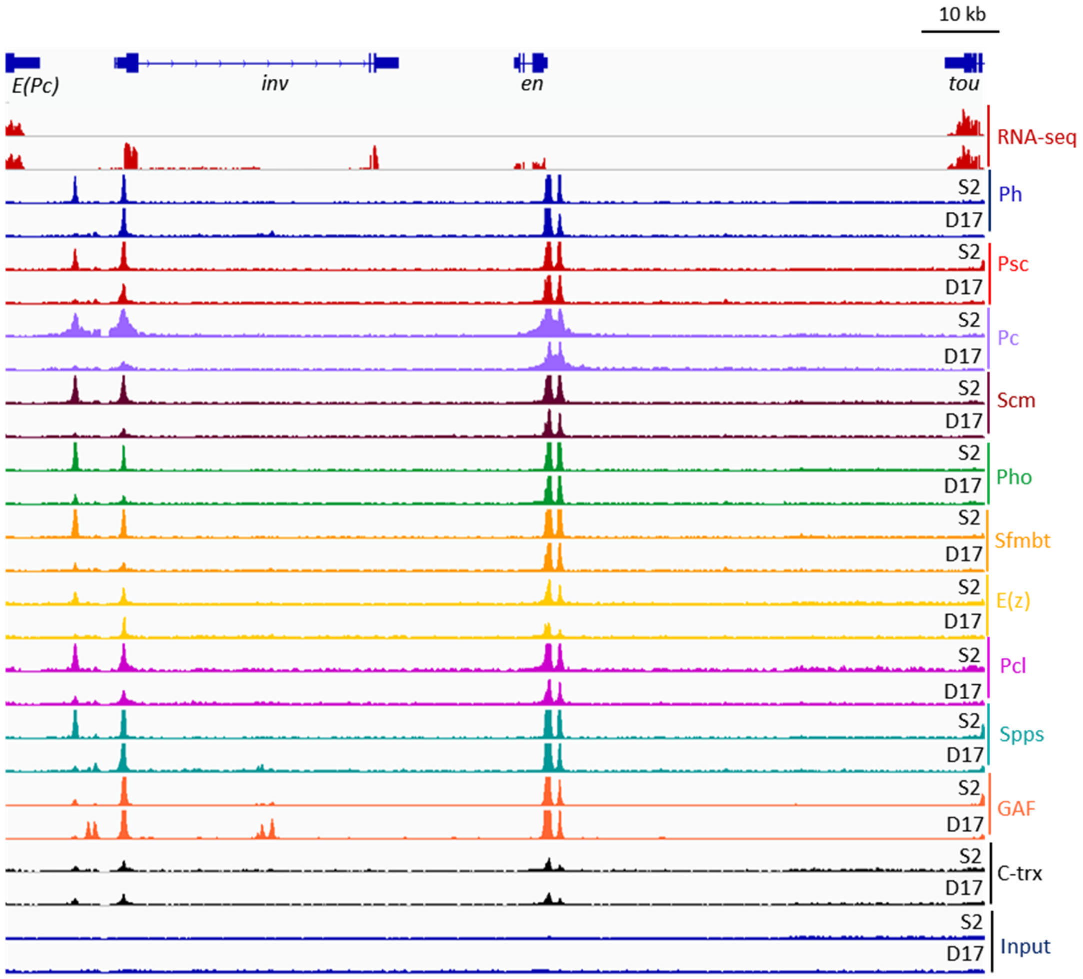

Fig. S2 Transcriptional pattern and the binding of various PcG proteins over the *inv-en* locus in S2 and D17 cells

The IGV tracks show the RNA-seq and normalized ChIP-seq data of various PcG proteins over the *inv-en* locus and the neighboring genes *E(Pc)* and *tou*. E(z), Pcl, C-trx and input are scaled at 0-7, and all other tracks are scaled at 0-10.

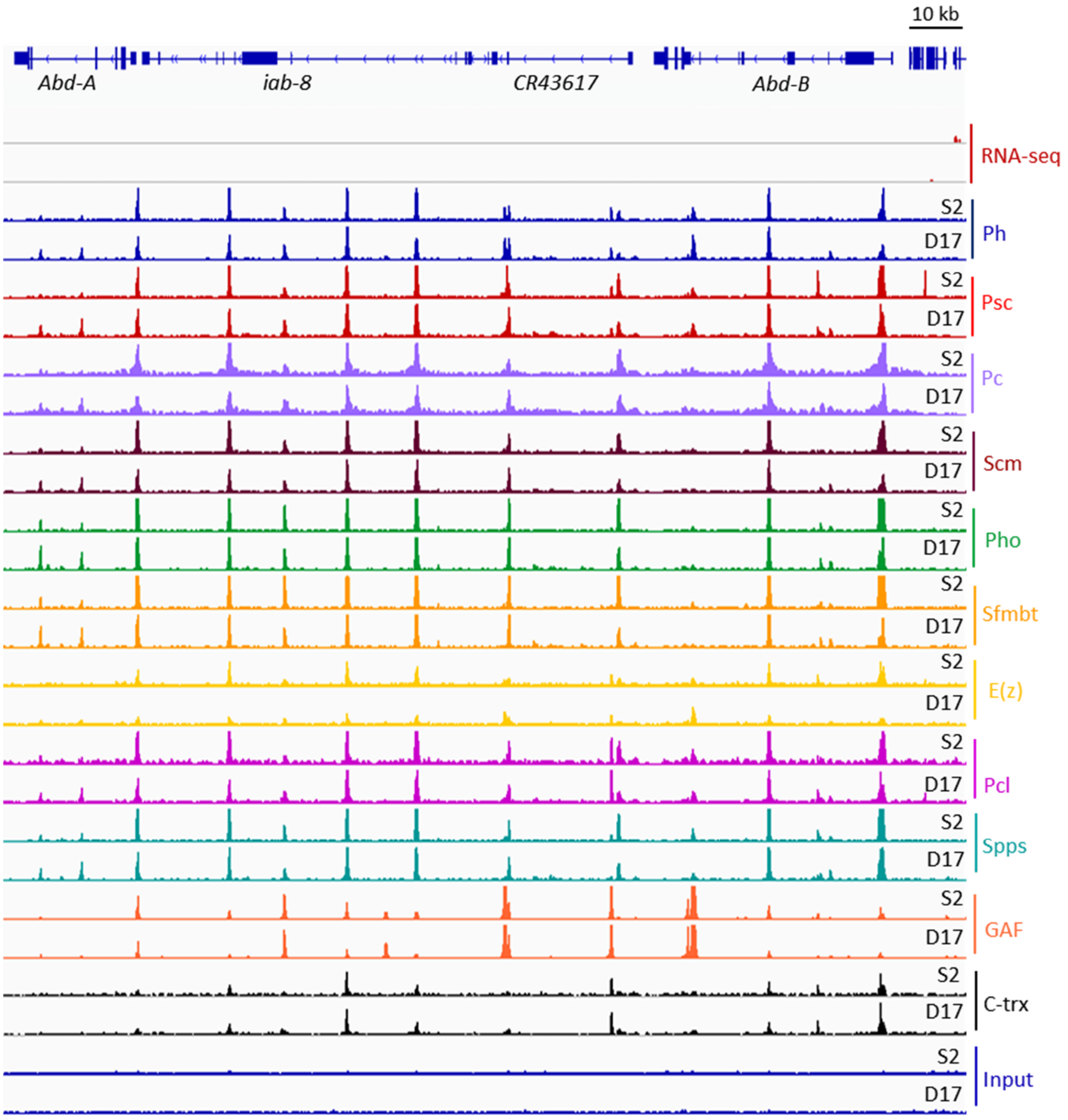

Fig. S3. Transcriptional pattern and the binding of various PcG proteins over the *abd-A/abd-B* region in S2 and D17 cells

The IGV tracks show the RNA-seq and normalized ChIP-seq data of various PcG proteins over the *abd-A* and *abd-B* region in S2 and D17 cells. E(z), Pcl, C-trx and input are scaled at 0-7, and all other tracks are scaled at 0-10.

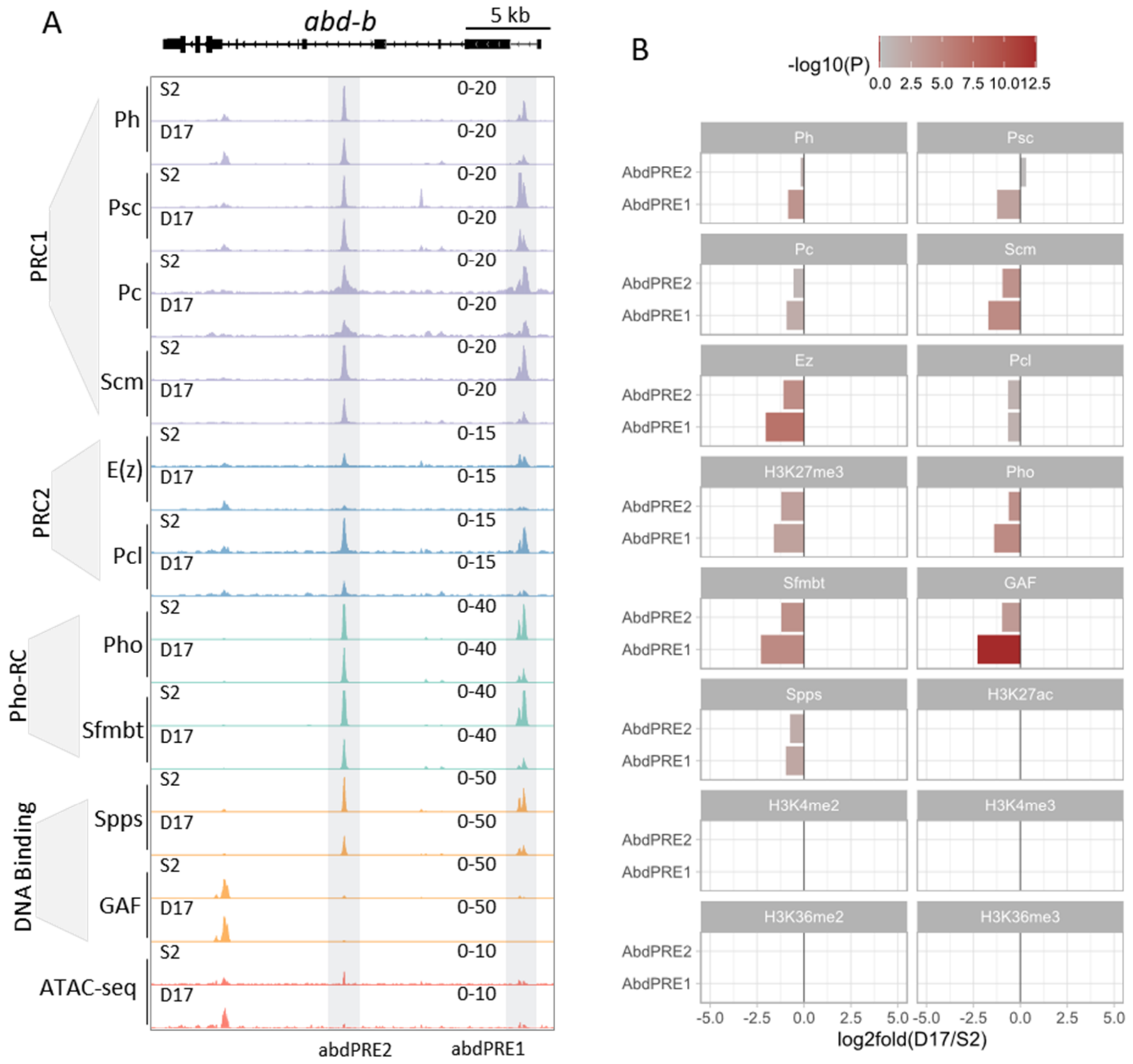

Fig. S4. Binding of core PcG and related proteins at *abd-b* PREs in S2 and D17 cells
This figure is related to the Fig. 2, except that it is for the *abd-b* locus. (A,B) IGV tracks show the occupancy of different PcG complex (PRC1, PRC2, Pho-RC), DNA binding factors and chromatin accessibility on the two *abd-b* PREs as control. (B) Bar plots show the differences in ChIP signals for different PcG proteins and related factors at each *abd-b* PREs between D17 and S2 cells.

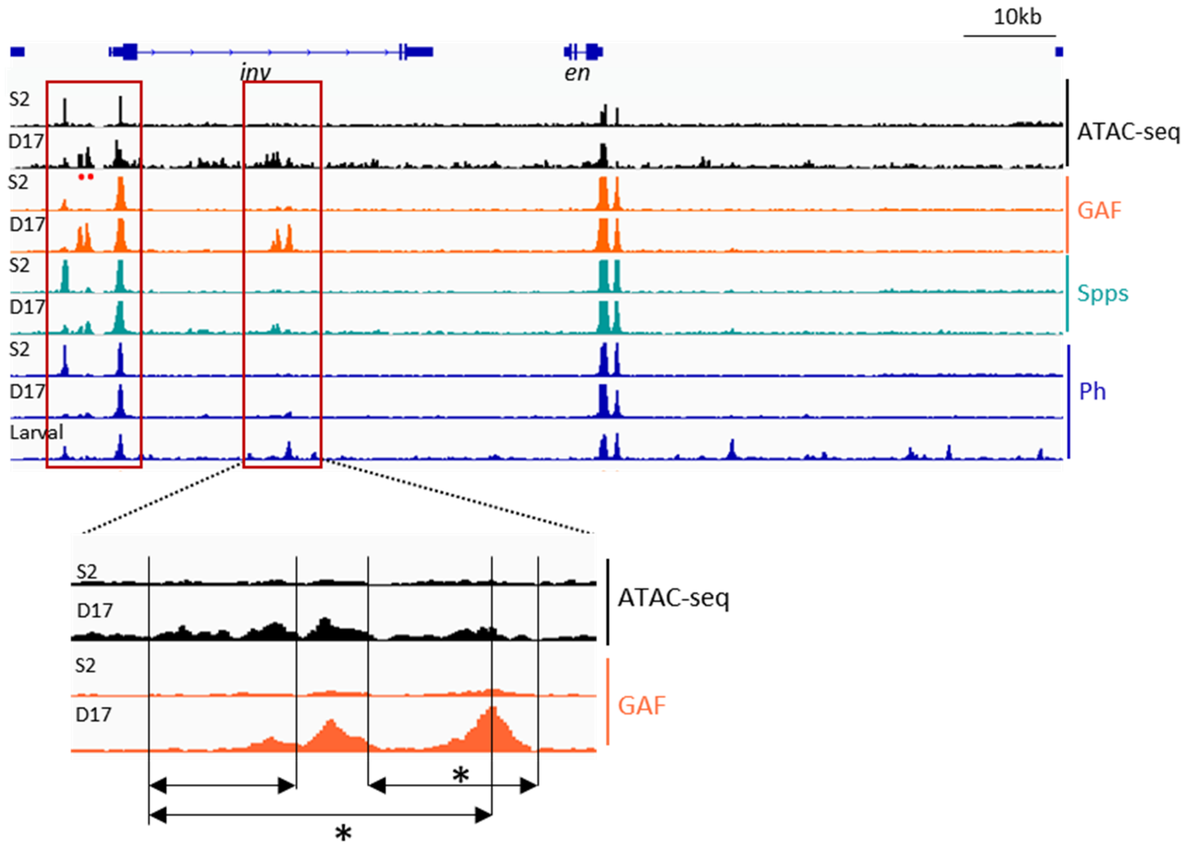

Fig. S5. ATAC-seq and ChIP-seq over *inv* and *en* in S2 and D17 cells

The red boxes highlight new binding peaks of GAF, Spps, and Ph and corresponding increased accessibility of chromatin in the ATAC-seq samples. The new peaks of binding within the *inv* gene are enlarged below. Three areas are highlighted by the black lines and double headed arrows to indicate fragments that were tested for enhancer activity by previous study (*54*). The two fragments marked by asterisks showed enhancer activity, both fragments contain one of the new GAF peaks and the increased accessibility of the chromatin. ATAC-seq is scaled at 0-5, GAF and Spps at 0-7, and Ph at 0-10.

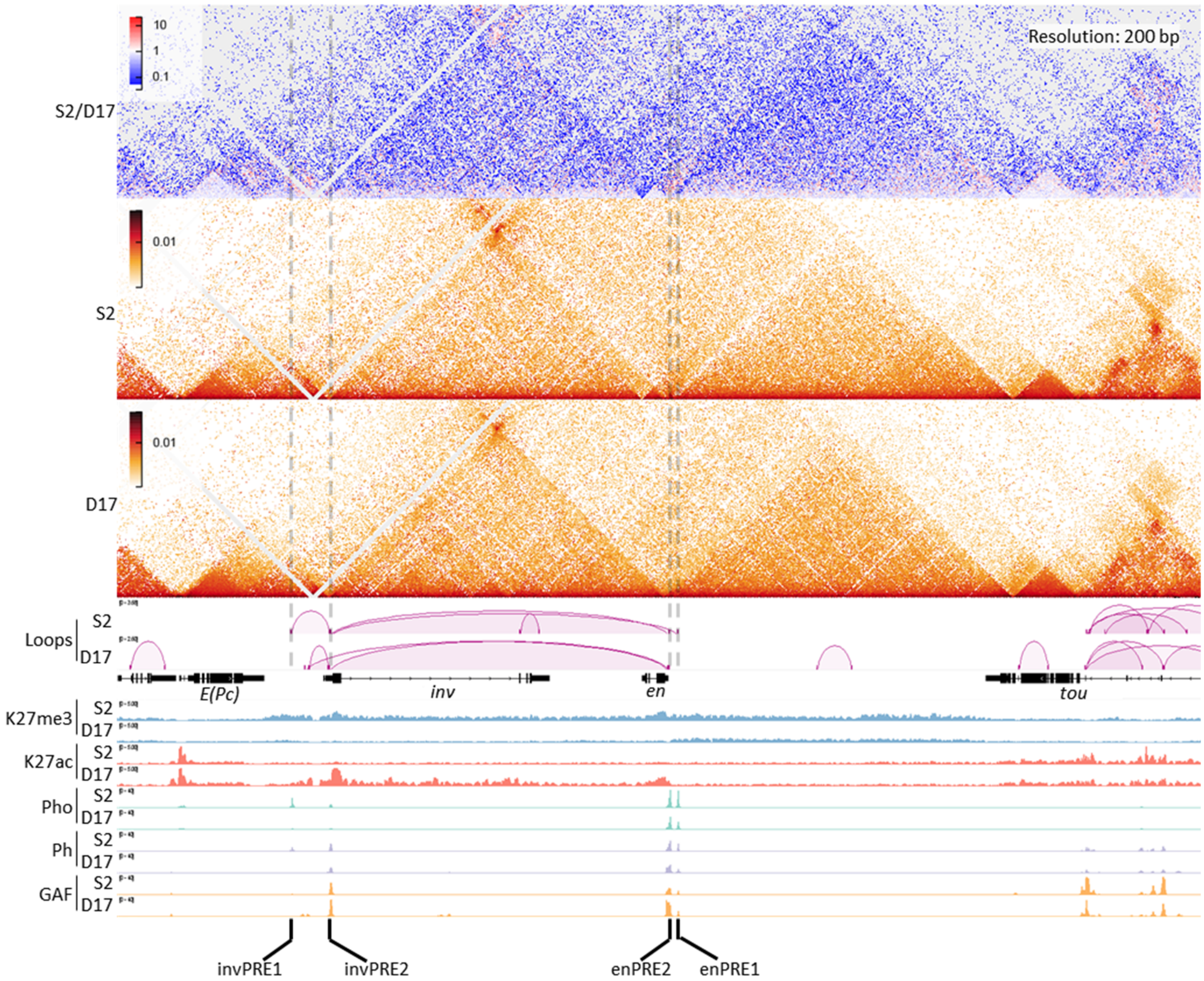

Fig. S6 PcG binding and chromatin structure over the *inv-en* domain in S2 and D17 cells This figure is related to Figure 3, except that here shows the large view. The contact map at top shows the differences between S2 and D17 cells, while the other two are for each cell type. The arrow shows the *en* transcription unit forms its own small domain in S2 cells, whereas it is in the same domain as *inv* in D17 cells. The significant loops are visualized as arcs just below the gene models, and the matched loop dots are also highlighted by green circles in the contact maps. At the bottom, the intensity for H3K27me3, H3K27ac, Pho, GAF and Ph are shown, with the D17-specific GAF binding sites highlighted in orange rectangles. The positions of the *inv-en* PREs are also indicated by dash lines and labelled at bottom.

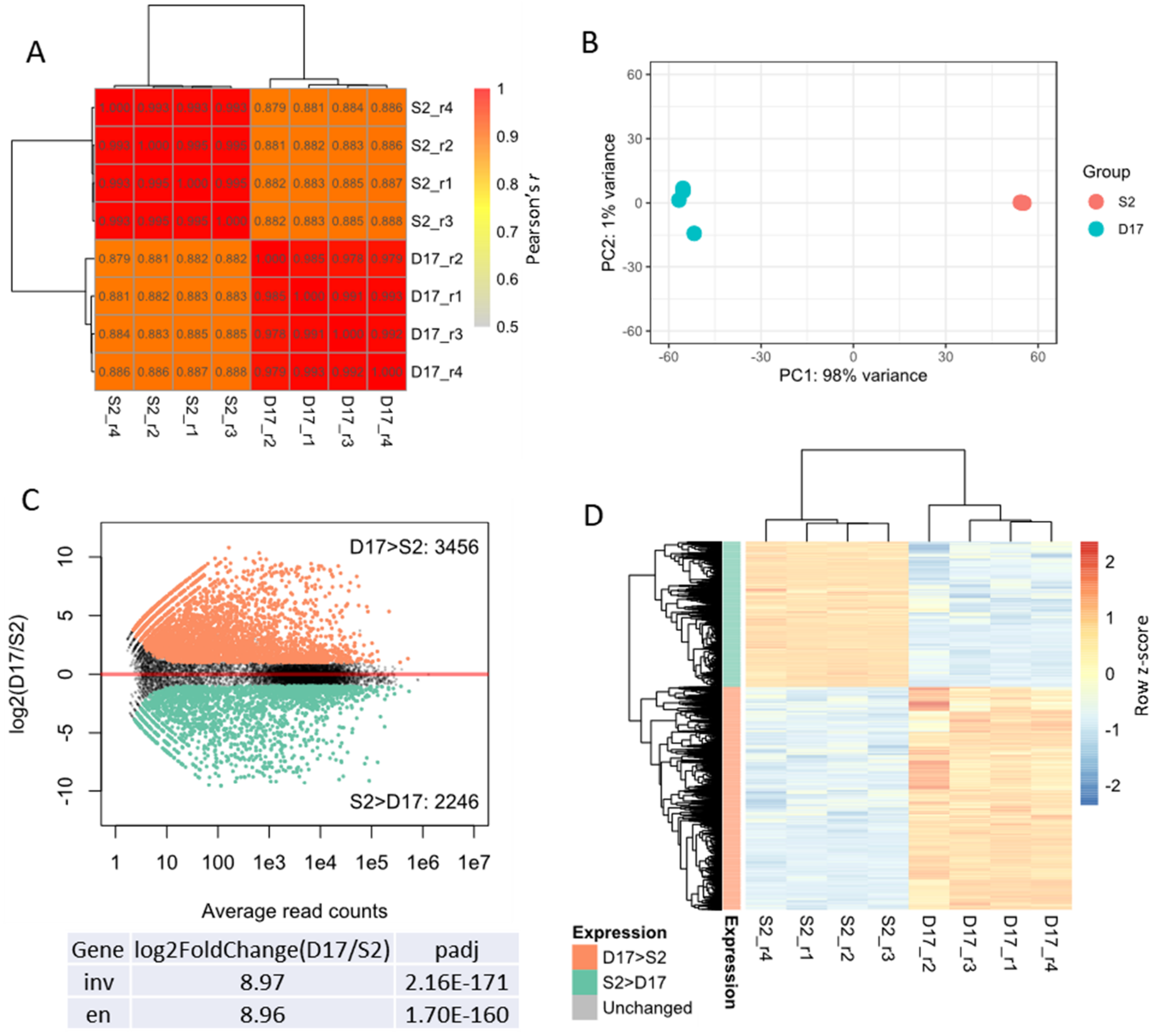

Gene	log2FoldChange(D17/S2)	padj
inv	8.97	2.16E-171
en	8.96	1.70E-160

Fig. S7. Differential gene expression between S2 and D17 cells

(**A**) Heatmap shows the clustering of different samples based on their transcriptomes. The color gradient indicates the Pearson's *r*. (**B**) PCA plot shows the relationship of different samples based on their transcriptomes. The top 500 genes with highest expression variation across samples were used. (**C**) MA plot shows the differentially expressed analysis result between the two cell types. (**D**) Heatmap shows the expression of the identified DEGs between S2 and D17 cells. The color gradient indicates the row z-score.

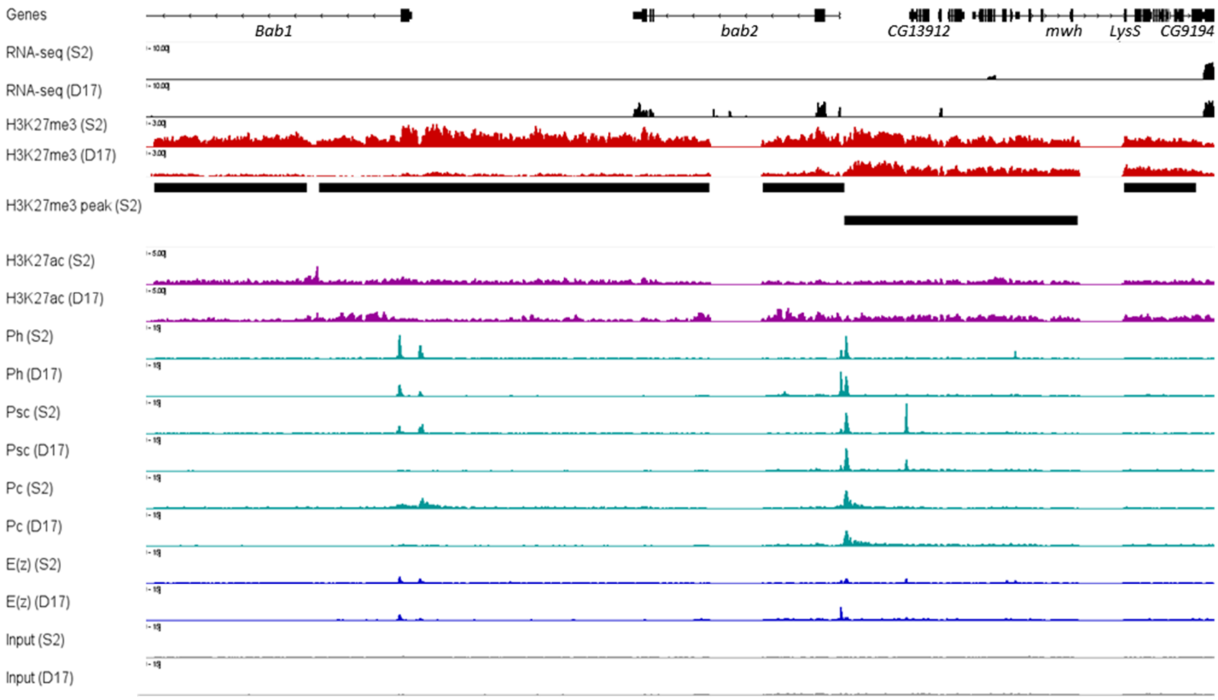

Region	Log2fold(D17/S2)	FDR
Subdomain-1	-13.6	2.8E-9
Subdomain-2	-0.7	0.00852

Fig. S8. Epigenetic and transcriptomic alterations of the subdomains at *bab1/bab2* locus between S2 and D17 cells

The IGV tracks show the RNA-seq data together with ChIP-seq data for H3K27me3, H3K27ac, Ph, Psc, Pc, E(z) at the *bab1/2* locus in S2 and D17 cells. The two subdomains that overlap *bab2* genes are labelled, and their DB analysis result is provided in the table below.

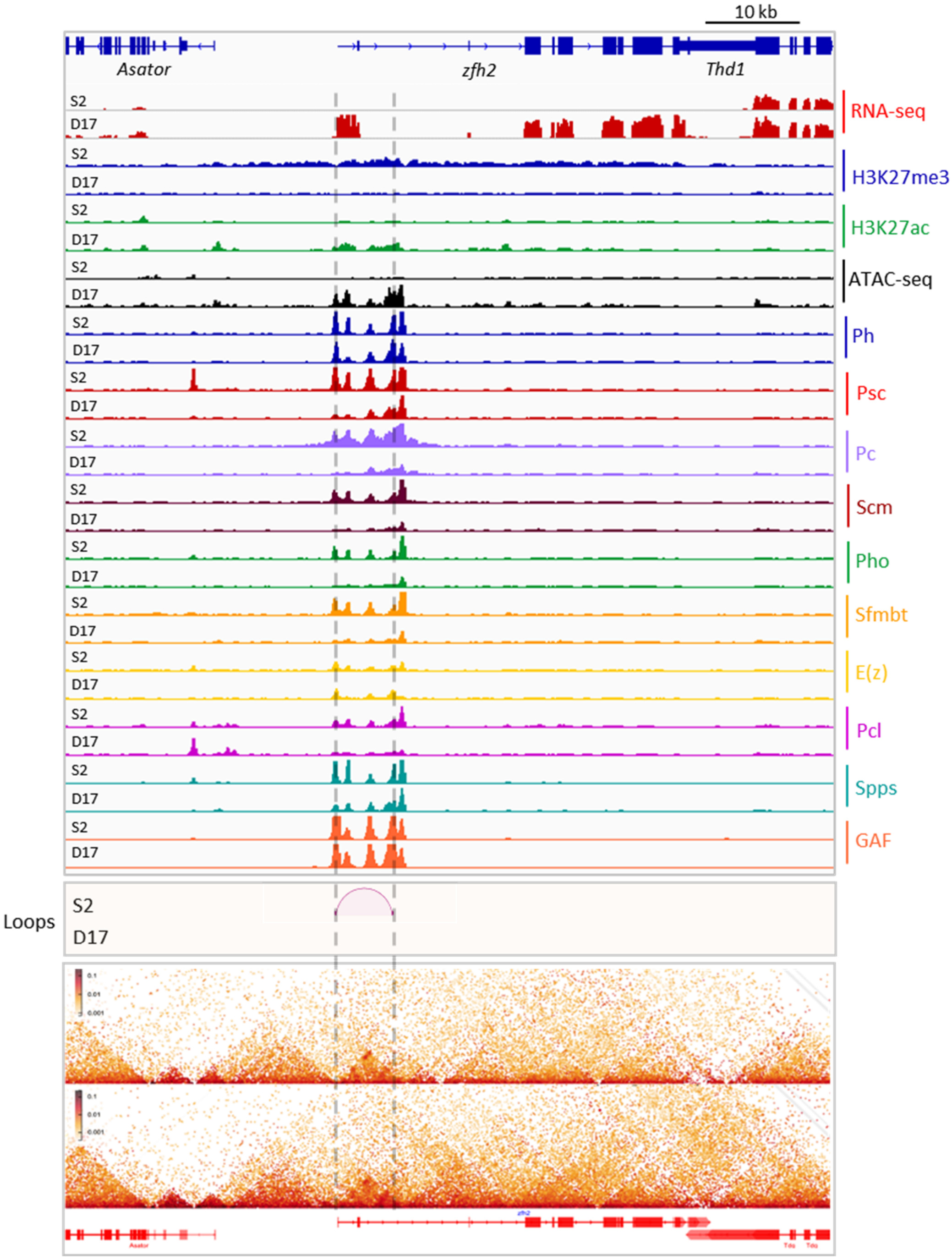

Fig. S9. Transcription, PcG protein binding and chromatin structure over the *zfh2* domain in S2 and D17 cells

The top panel shows RNA-seq, ATAC-seq and ChIP-seq data for *zfh2* domain in S2 and D17 cells. RNA-seq is scaled to 0-8, ATAC-seq, H3K27me3, H3K27ac, 0-5, Spps and GAF 0-10 and all other tracks at 0-7. The lower panel shows micro-C data over the same region in S2 and D17 cells at a 200 bp resolution.

Data S1. (separate file)
Sources of OMICs data used in this study.

Data S2. (separate file)
Differential binding analysis result of H3K27me3 between S2 and D17 cells.

Data S3. (separate file)
Chromatin loops identified from Micro-C data of S2 and D17 cells.

Data S4. (separate file)
Differentially expressed genes identified between S2 and D17 cells.

Data S5. (separate file)
List of antibodies used in this study.

REFERENCES AND NOTES

1. E. B. Lewis, A gene complex controlling segmentation in Drosophila. *Nature* **276**, 565–570 (1978).

2. B. Schuettengruber, H. M. Bourbon, L. Di Croce, G. Cavalli, Genome regulation by polycomb and trithorax: 70 years and counting. *Cell* **171**, 34–57 (2017).

3. M. I. Kuroda, H. Kang, S. De, J. A. Kassis, Dynamic competition of polycomb and trithorax in transcriptional programming. *Annu. Rev. Biochem.* **89**, 235–253 (2020).

4. J. A. Kassis, J. A. Kennison, J. W. Tamkun, Polycomb and trithorax group genes in Drosophila. *Genetics* **206**, 1699–1725 (2017).

5. J. J. Kim, R. E. Kingston, Context-specific Polycomb mechanisms in development. *Nat. Rev. Genet.*, (2022), **23**, 680, 695.

6. H. Kang, J. R. Cabrera, B. M. Zee, H. A. Kang, J. M. Jobe, M. B. Hegarty, A. E. Barry, A. Glotov, Y. B. Schwartz, M. I. Kuroda, Variant Polycomb complexes in Drosophila consistent with ancient functional diversity. *Sci. Adv.* **8**, eadd0103 (2022).

7. N. P. Blackledge, R. J. Klose, The molecular principles of gene regulation by Polycomb repressive complexes. *Nat. Rev. Mol. Cell Biol.* **22**, 815–833 (2021).

8. F. Frey, *T. Sheahan, K. Finkl, G. Stoehr, M. Mann, C. Benda, J. Müller*, Molecular basis of PRC1 targeting to Polycomb response elements by PhoRC. *Genes Dev.* **30**, 1116–1127 (2016).

9. J. C. Scheuermann, A. G. de Ayala Alonso, K. Oktaba, N. Ly-Hartig, R. K. McGinty, S. Fraterman, M. Wilm, T. W. Muir, J. Müller, Histone H2A deubiquitinase activity of the Polycomb repressive complex PR-DUB. *Nature* **465**, 243–247 (2010).

10. T. Klymenko, B. Papp, W. Fischle, T. Köcher, M. Schelder, C. Fritsch, B. Wild, M. Wilm, J. Müller, A Polycomb group protein complex with sequence-specific DNA-binding and selective methyl-lysine-binding activities. *Genes Dev.* **20**, 1110–1122 (2006).

11. J. A. Kassis, J. L. Brown, Polycomb group response elements in Drosophila and vertebrates. *Adv. Genet.* **81**, 83–118 (2013).

12. J. L. Brown, M. A. Sun, J. A. Kassis, Global changes of H3K27me3 domains and Polycomb group protein distribution in the absence of recruiters Spps or Pho. *Proc. Natl. Acad. Sci. U.S.A.* **115**, E1839-E1848 (2018).

13. B. M. Owen, C. Davidovich, DNA binding by polycomb-group proteins: Searching for the link to CpG islands. *Nucleic Acids Res.* **50**, 4813–4839 (2022).

14. J. I. Barrasa, T. G. Kahn, M. J. Lundkvist, Y. B. Schwartz, DNA elements tether canonical Polycomb repressive complex 1 to human genes. *Nucleic Acids Res.* **51**, 11613–11633 (2023).

15. E. M. Riising, I. Comet, B. Leblanc, X. Wu, J. V. Johansen, K. Helin, Gene silencing triggers polycomb repressive complex 2 recruitment to CpG islands genome wide. *Mol. Cell* **55**, 347–360 (2014).

16. A. M. Deaton, A. Bird, CpG islands and the regulation of transcription. *Genes Dev.* **25**, 1010–1022 (2011).

17. T. Pachano, V. Sánchez-Gaya, T. Ealo, M. Mariner-Faulí, T. Bleckwehl, H. G. Asenjo, P. Respuela, S. Cruz-Molina, M. Muñoz-San Martín, E. Haro, W. F. J. van IJcken, D. Landeira, A. Rada-Iglesias, Orphan CpG islands amplify poised enhancer regulatory activity and determine target gene responsiveness. *Nat. Genet.* **53**, 1036–1049 (2021).

18. L. Ringrose, R. Paro, Polycomb/trithorax response elements and epigenetic memory of cell identity. *Development* **134**, 223–232 (2007).

19. Y. B. Schwartz, T. G. Kahn, P. Stenberg, K. Ohno, R. Bourgon, V. Pirrotta, Alternative epigenetic chromatin states of polycomb target genes. *PLOS Genet.* **6**, e1000805 (2010).

20. B. Papp, J. Muller, Histone trimethylation and the maintenance of transcriptional ON and OFF states by trxG and PcG proteins. *Genes Dev.* **20**, 2041–2054 (2006).

21. Y. Ogiyama, B. Schuettengruber, G. L. Papadopoulos, J. M. Chang, G. Cavalli, Polycomb-dependent chromatin looping contributes to gene silencing during Drosophila development. *Mol. Cell* **71**, 73–88.e5 (2018).

22. V. Loubiere, G. L. Papadopoulos, Q. Szabo, A. M. Martinez, G. Cavalli, Widespread activation of developmental gene expression characterized by PRC1-dependent chromatin looping. *Sci. Adv.* **6**, eaax4001 (2020).

23. T. Nagano, Y. Lubling, T. J. Stevens, S. Schoenfelder, E. Yaffe, W. Dean, E. D. Laue, A. Tanay, P. Fraser, Single-cell Hi-C reveals cell-to-cell variability in chromosome structure. *Nature* **502**, 59–64 (2013).

24. A. Hafner, A. Boettiger, The spatial organization of transcriptional control. *Nat. Rev. Genet.* **24**, 53–68 (2023).

25. M. Levo, J. Raimundo, X. Y. Bing, Z. Sisco, P. J. Batut, S. Ryabichko, T. Gregor, M. S. Levine, Transcriptional coupling of distant regulatory genes in living embryos. *Nature* **605**, 754–760 (2022).

26. P. J. Batut, X. Y. Bing, Z. Sisco, J. Raimundo, M. Levo, M. S. Levine, Genome organization controls transcriptional dynamics during development. *Science* **375**, 566–570 (2022).

27. D. Kwon, D. Mucci, K. K. Langlais, J. L. Americo, S. K. DeVido, Y. Cheng, J. A. Kassis, Enhancer-promoter communication at the Drosophila engrailed locus. *Development* **136**, 3067–3075 (2009).

28. Y. B. Schwartz, T. G. Kahn, D. A. Nix, X. Y. Li, R. Bourgon, M. Biggin, V. Pirrotta, Genome-wide analysis of Polycomb targets in Drosophila melanogaster. *Nat. Genet.* **38**, 700–705 (2006).

29. S. K. Bowman, A. M. Deaton, H. Domingues, P. I. Wang, R. I. Sadreyev, R. E. Kingston, W. Bender, H3K27 modifications define segmental regulatory domains in the Drosophila bithorax complex. *eLife* **3**, e02833 (2014).

30. C. Kwong, *B. Adryan, I. Bell, L. Meadows, S. Russell, J. R. Manak, R. White*, Stability and dynamics of polycomb target sites in Drosophila development. *PLOS Genet.* **4**, e1000178 (2008).

31. M. Erokhin, P. Elizar'ev, A. Parshikov, P. Schedl, P. Georgiev, D. Chetverina, Transcriptional read-through is not sufficient to induce an epigenetic switch in the silencing activity of Polycomb response elements. *Proc. Natl. Acad. Sci. U.S.A.* **112**, 14930–14935 (2015).

32. M. Erokhin, J. L. Brown, D. Lomaev, N. E. Vorobyeva, L. Zhang, L. V. Fab, M. Y. Mazina, I. V. Kulakovskiy, R. H. Ziganshin, P. Schedl, P. Georgiev, M. A. Sun, J. A. Kassis, D. Chetverina, Crol contributes to PRE-mediated repression and Polycomb group proteins recruitment in Drosophila. *Nucleic Acids Res.* **51**, 6087–6100 (2023).

33. J. L. Brown, J. A. Kassis, Spps, a Drosophila Sp1/KLF family member, binds to PREs and is required for PRE activity late in development. *Development* **137**, 2597–2602 (2010).

34. J. L. Brown, D. Mucci, M. Whiteley, M. L. Dirksen, J. A. Kassis, The Drosophila Polycomb group gene pleiohomeotic encodes a DNA binding protein with homology to the transcription factor YY1. *Mol. Cell* **1**, 1057–1064 (1998).

35. P. Ray, S. de, A. Mitra, K. Bezstarosti, J. A. A. Demmers, K. Pfeifer, J. A. Kassis, Combgap contributes to recruitment of Polycomb group proteins inDrosophila. *Proc. Natl. Acad. Sci. U.S.A.* **113**, 3826–3831 (2016).

36. J. Dejardin, G. Cavalli, Recruitment of Drosophila Polycomb group proteins to chromatin by DSP1. *Nature* **434**, 533–538 (2005).

37. G. A. Orsi, S. Kasinathan, K. T. Hughes, S. Saminadin-Peter, S. Henikoff, K. Ahmad, High-resolution mapping defines the cooperative architecture of Polycomb response elements. *Genome Res.* **24**, 809–820 (2014).

38. J. Americo, M. Whiteley, J. L. Brown, M. Fujioka, J. B. Jaynes, J. A. Kassis, A complex array of DNA-binding proteins required for pairing-sensitive silencing by a polycomb group response element from the Drosophila engrailed gene. *Genetics* **160**, 1561–1571 (2002).

39. M. D. Cunningham, J. L. Brown, J. A. Kassis, Characterization of the polycomb group response elements of the Drosophila melanogaster invected Locus. *Mol. Cell. Biol.* **30**, 820–828 (2010).

40. J. L. Brown, J. A. Kassis, Architectural and functional diversity of polycomb group response elements inDrosophila. *Genetics* **195**, 407–419 (2013).

41. J. L. Brown, J. D. Price, M. Erokhin, J. A. Kassis, Context-dependent role of Pho binding sites in Polycomb complex recruitment in Drosophila. *Genetics* **224**, (2023).

42. S. De, Y. Cheng, M. A. Sun, N. D. Gehred, J. A. Kassis, Structure and function of an ectopic Polycomb chromatin domain. *Sci. Adv.* **5**, eaau9739 (2019).

43. S. De, A. Mitra, Y. Cheng, K. Pfeifer, J. A. Kassis, Formation of a polycomb-domain in the absence of strong polycomb response elements. *PLOS Genet.* **12**, e1006200 (2016).

44. J. Erceg, T. Pakozdi, R. Marco-Ferreres, Y. Ghavi-Helm, C. Girardot, A. P. Bracken, E. E. M. Furlong, Dual functionality ofcis-regulatory elements as developmental enhancers and Polycomb response elements. *Genes Dev.* **31**, 590–602 (2017).

45. L. Cherbas, Unpublished cell lines from the Miyake lab. *FBrf0205935*, (2008).

46. Y. Cheng, A. L. Brunner, S. Kremer, S. K. DeVido, C. M. Stefaniuk, J. A. Kassis, Co-regulation of invected and engrailed by a complex array of regulatory sequences in Drosophila. *Dev. Biol.* **395**, 131–143 (2014).

47. H. Kang, K. A. McElroy, Y. L. Jung, A. A. Alekseyenko, B. M. Zee, P. J. Park, M. I. Kuroda, Sex comb on midleg (Scm) is a functional link between PcG-repressive complexes in Drosophila. *Genes Dev.* **29**, 1136–1150 (2015).

48. K. K. Langlais, J. L. Brown, J. A. Kassis, Polycomb group proteins bind an engrailed PRE in both the "ON" and "OFF" transcriptional states of engrailed. *PLOS ONE* **7**, e48765 (2012).

49. N. Soshnikova, D. Duboule, Epigenetic temporal control of mouse Hox genes in vivo. *Science* **324**, 1320–1323 (2009).

50. H. K. Long, S. L. Prescott, J. Wysocka, Ever-changing landscapes: Transcriptional enhancers in development and evolution. *Cell* **167**, 1170–1187 (2016).

51. S. L. Klemm, Z. Shipony, W. J. Greenleaf, Chromatin accessibility and the regulatory epigenome. *Nat. Rev. Genet.* **20**, 207–220 (2019).

52. G. Hunt, A. Boija, M. p300/CBP sustains Polycomb silencing by non-enzymatic functions. *Mol. Cell* **82**, 3580–3597.e9 (2022).

53. P. V. Kharchenko, A. A. Alekseyenko, Y. B. Schwartz, A. Minoda, N. C. Riddle, J. Ernst, P. J. Sabo, E. Larschan, A. A. Gorchakov, T. Gu, D. Linder-Basso, A. Plachetka, G. Shanower, M. Y. Tolstorukov, L. J. Luquette, R. Xi, Y. L. Jung, R. W. Park, E. P. Bishop, T. K. Canfield, R. Sandstrom, R. E. Thurman, D. M. MacAlpine, J. A. Stamatoyannopoulos, M. Kellis, S. C. R. Elgin, M. I. Kuroda, V. Pirrotta, G. H. Karpen, P. J. Park, Comprehensive analysis of the chromatin landscape in Drosophila melanogaster. *Nature* **471**, 480–485 (2011).

54. K. Gurdziel, D. S. Lorberbaum, A. M. Udager, J. Y. Song, N. Richards, D. S. Parker, L. A. Johnson, B. L. Allen, S. Barolo, D. L. Gumucio, Identification and validation of novel hedgehog-responsive enhancers predicted by computational analysis of Ci/Gli binding site density. *PLOS ONE* **10**, e0145225 (2015).

55. X. Li, X. Tang, X. Bing, C. Catalano, T. Li, G. Dolsten, C. Wu, M. Levine, GAGA-associated factor fosters loop formation in the Drosophila genome. *Mol. Cell*, (2023), **83**, 1519, 1526.e4.

56. I. F. Davidson, J. M. Peters, Genome folding through loop extrusion by SMC complexes. *Nat. Rev. Mol. Cell Biol.* **22**, 445–464 (2021).

57. K. P. Eagen, E. L. Aiden, R. D. Kornberg, Polycomb-mediated chromatin loops revealed by a subkilobase-resolution chromatin interaction map. *Proc. Natl. Acad. Sci. U.S.A.* **114**, 8764–8769 (2017).

58. C. Y. Ngan, C. H. Wong, H. Tjong, W. Wang, R. L. Goldfeder, C. Choi, H. He, L. Gong, J. Lin, B. Urban, J. Chow, M. Li, J. Lim, V. Philip, S. A. Murray, H. Wang, C. L. Wei, Chromatin interaction analyses elucidate the roles of PRC2-bound silencers in mouse development. *Nat. Genet.* **52**, 264–272 (2020).

59. R. Rickels, D. Hu, C. K. Collings, A. R. Woodfin, A. Piunti, M. Mohan, H. M. Herz, E. Kvon, A. Shilatifard, An evolutionary conserved epigenetic mark of polycomb response elements implemented by Trx/MLL/COMPASS. *Mol. Cell* **63**, 318–328 (2016).

60. H. Kang, Y. L. Jung, K. A. McElroy, B. M. Zee, H. A. Wallace, J. L. Woolnough, P. J. Park, M. I. Kuroda, Bivalent complexes of PRC1 with orthologs of BRD4 and MOZ/MORF target developmental genes in Drosophila. *Genes Dev.* **31**, 1988–2002 (2017).

61. G. Strubbe, Polycomb purification by in vivo biotinylation tagging reveals cohesin and Trithorax group proteins as interaction partners. *Proc. Natl. Acad. Sci. U.S.A.* **108**, 5572–5577 (2011).

62. C. A. Schaaf, Z. Misulovin, M. Gause, A. Koenig, D. W. Gohara, A. Watson, D. Dorsett, Cohesin and polycomb proteins functionally interact to control transcription at silenced and active genes. *PLOS Genet.* **9**, e1003560 (2013).

63. K. T. Chathoth, N. R. Zabet, Chromatin architecture reorganization during neuronal cell differentiation inDrosophilagenome. *Genome Res.* **29**, 613–625 (2019).

64. C. A. Schaaf, Z. Misulovin, G. Sahota, A. M. Siddiqui, Y. B. Schwartz, T. G. Kahn, V. Pirrotta, M. Gause, D. Dorsett, Regulation of the Drosophila Enhancer of split and invected-engrailed gene complexes by sister chromatid cohesion proteins. *PLOS ONE* **4**, e6202 (2009).

65. Y. Cheng, F. Chan, J. A. Kassis, The activity of engrailed imaginal disc enhancers is modulated epigenetically by chromatin and autoregulation. *PLOS Genet.* **19**, e1010826 (2023).

66. B. Langmead, S. L. Salzberg, Fast gapped-read alignment with Bowtie 2. *Nat. Methods* **9**, 357–359 (2012).

67. H. Li, B. Handsaker, A. Wysoker, T. Fennell, J. Ruan, N. Homer, G. Marth, G. Abecasis, R. Durbin, 1000 Genome Project Data Processing Subgroup, The Sequence Alignment/Map format and SAMtools. *Bioinformatics* **25**, 2078–2079 (2009).

68. Y. Zhang, T. Liu, C. A. Meyer, J. Eeckhoute, D. S. Johnson, B. E. Bernstein, C. Nusbaum, R. M. Myers, M. Brown, W. Li, X. S. Liu, Model-based analysis of ChIP-Seq (MACS). *Genome Biol.* **9**, R137 (2008).

69. H. M. Amemiya, A. Kundaje, A. P. Boyle, The ENCODE Blacklist: Identification of problematic regions of the genome. *Sci. Rep.* **9**, 9354 (2019).

70. C. S. Ross-Innes, R. Stark, A. E. Teschendorff, K. A. Holmes, H. R. Ali, M. J. Dunning, G. D. Brown, O. Gojis, I. O. Ellis, A. R. Green, S. Ali, S. F. Chin, C. Palmieri, C. Caldas, J. S. Carroll, Differential oestrogen receptor binding is associated with clinical outcome in breast cancer. *Nature* **481**, 389–393 (2012).

71. A. Dobin, C. A. Davis, F. Schlesinger, J. Drenkow, C. Zaleski, S. Jha, P. Batut, M. Chaisson, T. R. Gingeras, STAR: Ultrafast universal RNA-seq aligner. *Bioinformatics* **29**, 15–21 (2013).

72. Y. Liao, G. K. Smyth, W. Shi, The Subread aligner: Fast, accurate and scalable read mapping by seed-and-vote. *Nucleic Acids Res.* **41**, e108 (2013).

73. M. I. Love, W. Huber, S. Anders, Moderated estimation of fold change and dispersion for RNA-seq data with DESeq2. *Genome Biol.* **15**, 550 (2014).

74. B. Li, C. N. Dewey, RSEM: Accurate transcript quantification from RNA-Seq data with or without a reference genome. *BMC Bioinformatics* **12**, 323 (2011).

75. E. Slobodyanyuk, C. Cattoglio, T. S. Hsieh, Mapping mammalian 3D genomes by micro-C. *Methods Mol. Biol.* **2532**, 51–71 (2022).

76. S. Chakraborty, N. Kopitchinski, Z. Zuo, A. Eraso, P. Awasthi, R. Chari, A. Mitra, I. C. Tobias, S. D. Moorthy, R. K. Dale, J. A. Mitchell, T. J. Petros, P. P. Rocha, Enhancer-promoter interactions can bypass CTCF-mediated boundaries and contribute to phenotypic robustness. *Nat. Genet.*, (2023), **55**, 280, 290.

77. N. Servant, N. Varoquaux, B. R. Lajoie, E. Viara, C. J. Chen, J. P. Vert, E. Heard, J. Dekker, E. Barillot, HiC-Pro: An optimized and flexible pipeline for Hi-C data processing. *Genome Biol.* **16**, 259 (2015).

78. P. Kerpedjiev, N. Abdennur, F. Lekschas, C. McCallum, K. Dinkla, H. Strobelt, J. M. Luber, S. B. Ouellette, A. Azhir, N. Kumar, J. Hwang, S. Lee, B. H. Alver, H. Pfister, L. A. Mirny, P. J. Park, N. Gehlenborg, HiGlass: Web-based visual exploration and analysis of genome interaction maps. *Genome Biol.* **19**, 125 (2018).

79. N. Abdennur, L. A. Mirny, Cooler: Scalable storage for Hi-C data and other genomically labeled arrays. *Bioinformatics* **36**, 311–316 (2020).

80. A. Roayaei Ardakany, H. T. Gezer, S. Lonardi, F. Ay, Mustache: Multi-scale detection of chromatin loops from Hi-C and Micro-C maps using scale-space representation. *Genome Biol.* **21**, 256 (2020).

81. A. D. Yates, Ensembl 2020. *Nucleic Acids Res.* **48**, D682-D688 (2019).

82. R. C. Team, R: A language and environment for statistical computing. *R Foundation for Statistical Computing, Vienna, Austria.*, (2020).

83. F. Ramirez, F. Dundar, S. Diehl, B. A. Gruning, T. Manke, deepTools: A flexible platform for exploring deep-sequencing data. *Nucleic Acids Res.* **42**, W187–191 (2014).

84. H. Thorvaldsdottir, J. T. Robinson, J. P. Mesirov, Integrative Genomics Viewer (IGV): High-performance genomics data visualization and exploration. *Brief. Bioinform.* **14**, 178–192 (2013).

85. C. Albig, *E. Tikhonova, S. Krause, O. Maksimenko, C. Regnard, P. B. Becker,* Factor cooperation for chromosome discrimination inDrosophila. *Nucleic Acids Res.* **47**, 1706–1724 (2019).

86. P. Buchenau, J. Hodgson, H. Strutt, D. J. Arndt-Jovin, The distribution of polycomb-group proteins during cell division and development in Drosophila embryos: Impact on models for silencing. *J. Cell Biol.* **141**, 469–481 (1998).

87. N. J. Francis, N. E. Follmer, M. D. Simon, G. Aghia, J. D. Butler, Polycomb proteins remain bound to chromatin and DNA during DNA replication in vitro. *Cell* **137**, 110–122 (2009).

88. C. Grimm, R. Matos, N. Ly-Hartig, U. Steuerwald, D. Lindner, V. Rybin, J. Müller, *C. W. Müller,* Molecular recognition of histone lysine methylation by the Polycomb group repressor dSfmbt. *EMBO J.* **28**, 1965–1977 (2009).

89. N. Negre, A cis-regulatory map of the Drosophila genome. *Nature* **471**, 527–531 (2011).

90. J. L. Brown, C. Fritsch, J. Mueller, J. A. Kassis, TheDrosophila pho-likegene encodes a YY1-related DNA binding protein that is redundant withpleiohomeoticin homeotic gene silencing. *Development* **130**, 285–294 (2003)
